# Intermittent Preventive Treatment of Malaria in Pregnancy with Mefloquine in HIV-Infected Women Receiving Cotrimoxazole Prophylaxis: A Multicenter Randomized Placebo-Controlled Trial

**DOI:** 10.1371/journal.pmed.1001735

**Published:** 2014-09-23

**Authors:** Raquel González, Meghna Desai, Eusebio Macete, Peter Ouma, Mwaka A. Kakolwa, Salim Abdulla, John J. Aponte, Helder Bulo, Abdunoor M. Kabanywanyi, Abraham Katana, Sonia Maculuve, Alfredo Mayor, Arsenio Nhacolo, Kephas Otieno, Golbahar Pahlavan, María Rupérez, Esperança Sevene, Laurence Slutsker, Anifa Vala, John Williamsom, Clara Menéndez

**Affiliations:** 1 Barcelona Centre for International Health Research (CRESIB, Hospital Clínic-Universitat de Barcelona), ISGlobal, Barcelona Institute for Global Health, Barcelona, Spain; 2 Manhiça Health Research Center (CISM), Manhiça, Mozambique; 3 Kenya Medical Research Institute/Centers for Disease Control and Prevention (KEMRI/CDC) Research and Public Health Collaboration, Kisumu, Kenya; 4 Division of Parasitic Diseases and Malaria, Center for Global Health, Centers for Disease Control and Prevention, Atlanta, Georgia, United States of America, and Kisumu, Kenya; 5 Kenya Medical Research Institute (KEMRI)/Center for Global Health Research, Kisumu, Kenya; 6 Ifakara Health Institute (IHI), Dodoma, Tanzania; National Institute of Child Health and Human Development, United States of America

## Abstract

**Background:**

Intermittent preventive treatment in pregnancy (IPTp) with sulfadoxine-pyrimethamine (SP) is recommended for malaria prevention in HIV-negative pregnant women, but it is contraindicated in HIV-infected women taking daily cotrimoxazole prophylaxis (CTXp) because of potential added risk of adverse effects associated with taking two antifolate drugs simultaneously. We studied the safety and efficacy of mefloquine (MQ) in women receiving CTXp and long-lasting insecticide treated nets (LLITNs).

**Methods and Findings:**

A total of 1,071 HIV-infected women from Kenya, Mozambique, and Tanzania were randomized to receive either three doses of IPTp-MQ (15 mg/kg) or placebo given at least one month apart; all received CTXp and a LLITN. IPTp-MQ was associated with reduced rates of maternal parasitemia (risk ratio [RR], 0.47 [95% CI 0.27–0.82]; *p* = 0.008), placental malaria (RR, 0.52 [95% CI 0.29–0.90]; *p* = 0.021), and reduced incidence of non-obstetric hospital admissions (RR, 0.59 [95% CI 0.37–0.95]; *p* = 0.031) in the intention to treat (ITT) analysis. There were no differences in the prevalence of adverse pregnancy outcomes between groups. Drug tolerability was poorer in the MQ group compared to the control group (29.6% referred dizziness and 23.9% vomiting after the first IPTp-MQ administration). HIV viral load at delivery was higher in the MQ group compared to the control group (*p* = 0.048) in the ATP analysis. The frequency of perinatal mother to child transmission of HIV was increased in women who received MQ (RR, 1.95 [95% CI 1.14–3.33]; *p* = 0.015). The main limitation of the latter finding relates to the exploratory nature of this part of the analysis.

**Conclusions:**

An effective antimalarial added to CTXp and LLITNs in HIV-infected pregnant women can improve malaria prevention, as well as maternal health through reduction in hospital admissions. However, MQ was not well tolerated, limiting its potential for IPTp and indicating the need to find alternatives with better tolerability to reduce malaria in this particularly vulnerable group. MQ was associated with an increased risk of mother to child transmission of HIV, which warrants a better understanding of the pharmacological interactions between antimalarials and antiretroviral drugs.

**Trial registration:**

ClinicalTrials.gov NCT 00811421; Pan African Clinical Trials Registry PACTR 2010020001813440

*Please see later in the article for the Editors' Summary*

## Abstract 2

Clara Menéndez and colleagues conducted a randomized controlled trial among HIV-positive pregnant women in Kenya, Mozambique, and Tanzania to investigate the safety and efficacy of mefloquine as intermittent preventative therapy for malaria in women receiving cotrimoxazole prophylaxis and long-lasting insecticide treated nets.

*Please see later in the article for the Editors' Summary*

## Editors' Summary

**Background:**

Malaria, a mosquito-borne parasitic disease, kills about 600,000 people every year. Most of these deaths occur among young children living in sub-Saharan Africa but pregnant women living in Africa are also very vulnerable to malaria. Infection with malaria during pregnancy can cause severe maternal anemia (reduced red blood cell numbers), stillbirths, and pre-term and low-birthweight babies, and is responsible for the deaths of many African women and their babies. To reduce the loss of life from malaria in pregnancy, the World Health Organization (WHO) recommends that pregnant women living in Africa receive the antimalarial drug sulfadoxine-pyrimethamine (SP) at each scheduled antenatal care visit given at least a month apart (intermittent preventive treatment in pregnancy [IPTp]). In addition, WHO advises pregnant women to sleep under insecticide-treated bed nets to protect themselves from the bites of infected mosquitoes and recommends effective case management of pregnant women with malarial illness.

**Why Was This Study Done?:**

Pregnant women living in Africa are often infected with HIV, the virus that causes AIDS. HIV infection increases both the risk and severity of malaria infection during pregnancy, and at least one million pregnancies are complicated by co-infection with malaria and HIV in sub-Saharan Africa every year. WHO recommends that HIV-positive pregnant women take cotrimoxazole (CTX) daily to prevent opportunistic infections (CTX prophylaxis [CTXp]). Unfortunately, both CTX and SP are antifolate drugs and taking two drugs of this type increases a woman's risk of developing a severe skin reaction. Moreover, although CTXp protects children and HIV-infected adults against malaria, it is not known whether CTXp alone protects HIV-infected pregnant women adequately against malaria. Thus, evaluations of alternative drugs for use in IPTp in HIV-positive pregnant women are needed. In this randomized placebo-controlled trial, the researchers study the safety and efficacy of the antimalarial drug mefloquine (MQ) in HIV-infected women receiving CTXp. A randomized, placebo-controlled trial compares outcomes among people chosen through the play of chance to receive either the drug under investigation or a “dummy” (placebo) drug.

**What Did the Researchers Do and Find?:**

The researchers allocated 1,071 HIV-infected pregnant women from Kenya, Mozambique, and Tanzania to receive three doses of MQ (IPTp-MQ), given at least one month apart, or three doses of placebo. All the women received CTXp and were given an insecticide-treated bed net. In an intention-to-treat analysis (an analysis that considers the outcomes of all trial participants irrespective of whether they receive their allocated treatment), the prevalence of parasitemia (parasites in the blood) at delivery among women given IPTp-MQ was 3.5% whereas the prevalence among women given the placebo was 6.9%. In other words, compared to placebo, IPTp-MQ was associated with a reduced risk of maternal parasitemia. IPTp-MQ was also associated with a reduced rate of placental malaria (parasites in the placenta) and a reduced incidence of hospital admissions for non-pregnancy related causes. There was no difference in adverse pregnancy outcomes such as stillbirth between the intervention groups but drug tolerability was poorer in the MQ group than in the placebo group. Finally, in an exploratory (unplanned) according-to-protocol analysis (an analysis that only considers outcomes in trial participants who receive their allocated intervention), women in the MQ group had a higher HIV viral load at delivery than women in the control group and were nearly twice as likely to transmit HIV to their child around the time of birth.

**What Do These Findings Mean?:**

These findings suggest that the addition of IPTp-MQ to CTXp and the use of insecticide-treated bed nets can improve malaria prevention and maternal health in HIV-infected pregnant women in Africa. However, the poor tolerability of MQ and the association of MQ treatment with both an increased HIV viral load at delivery and a higher frequency of mother-to-child-transmission of HIV when compared to placebo raise concerns about the use of MQ in IPTp. Because these last two findings came from an exploratory analysis, which is more likely to throw up a chance finding than a pre-planned analysis further studies are needed to confirm these unexpected but potentially important findings. Nevertheless, overall, the findings of this study suggest that MQ should not be recommended for IPTp in HIV-infected pregnant women in Africa and highlight the need to find alternative drugs for malaria prevention in this group of women who are particularly vulnerable to malaria.

**Additional Information:**

Please access these websites via the online version of this summary at http://dx.doi.org/10.1371/journal.pmed.1001735.

- This study is further discussed in a *PLOS Medicine* Perspective by Richard Steketee.
- A related *PLOS Medicine* Research Article by González et al. compares the efficacy of IPTp-MQ and IPTp-SP in HIV-negative women
- Information is available from the World Health Organization on malaria (in several languages) and on malaria in pregnancy; information on IPTp and the current WHO policy recommendation on IPTp with SP are available; the 2013 World Malaria Report provides details of the current global malaria situation
- The US Centers for Disease Control and Prevention also provides information on malaria; a personal story about malaria in pregnancy is available
- Information is available from the Roll Back Malaria Partnership on all aspects of global malaria control, including information on malaria in pregnancy
- The Malaria in Pregnancy Consortium is undertaking research into the prevention and treatment of malaria in pregnancy
- MedlinePlus provides links to additional information on malaria (in English and Spanish)
- More information about the trial protocol is available

## Introduction

Many African pregnant women are infected with HIV, and many of them are also exposed to falciparum malaria [1],[2]. It is estimated that at least 1 million pregnancies per year are complicated by co-infection of malaria and HIV in sub-Saharan Africa [3]. The interaction between malaria and HIV is particularly deleterious in pregnant women, leading to increased risk and severity of both malaria infection and disease [1],[2]. In addition, HIV infection attenuates the relative protection against malaria acquired with subsequent pregnancies, placing more women at risk for malaria-related complications [4],[5]. Moreover, HIV infection reduces the efficacy of malaria interventions and complicates the use of antimalarials because of potential drug interactions [6],[7].

In Africa, intermittent preventive treatment in pregnancy (IPTp) with sulfadoxine-pyrimethamine (SP) is recommended monthly at each antenatal care (ANC) visit, along with the use of insecticide treated nets, for malaria prevention in HIV-negative pregnant women [8]. In areas where HIV prevalence among pregnant women was higher than 10%, the WHO recommended at least three IPTp-SP doses [9]. Moreover, in HIV-infected pregnant women living in areas with limited health resources and high HIV prevalence, universal cotrimoxazole prophylaxis (CTXp) is recommended to prevent opportunistic infections [10]. Since the risk of severe cutaneous reactions is increased in individuals taking CTXp and SP concomitantly, and especially in those who are HIV-infected, IPTp with SP is contraindicated in HIV-infected pregnant women on CTXp [11],[12]. In addition, CTXp has been shown to protect against malaria infection and illness in children [13] and in HIV-infected adults [14]. However, the latter has not been adequately studied in pregnant HIV-infected women. Thus, it is important to evaluate whether provision of an antimalarial as IPTp in addition to CTXp is needed to provide the most efficacious malaria prevention to the most vulnerable pregnant women, those who are infected with HIV.

Since SP cannot be evaluated in individuals on CTXp, other antimalarials for IPTp should be considered. Candidate antimalarials for IPTp should ideally have three main attributes: a long half-life to maximize the prophylactic effect; be administered in single dose to ensure adherence; and have a proven acceptable reproductive toxicity safety profile. [11],[12]. Among available antimalarial drugs, mefloquine (MQ) offers the most comparative advantages to SP for IPTp. This long-acting antimalarial (half-life approximately 2–3 weeks) retains high efficacy in Africa [15]–[17], and is considered safe throughout pregnancy as evidenced by its recommendation by the WHO and the US Centers for Disease Control and Prevention (CDC) for chemoprophylaxis for pregnant women of all gestational ages travelling in malaria endemic regions. MQ has also been recently reclassified as pregnancy category B by the US Food and Drug Administration [18],[19]. Notably, MQ has been reported to be associated with poor tolerability, with frequent vomiting and dizziness, as well as mild neurological and psychological adverse effects [20]. However, the existing information on the safety and efficacy of MQ as IPTp is limited in pregnant women, and even more so in those who are HIV-infected [21]–[23].

We report here the results of a multicenter double-blind placebo-controlled randomized trial conducted in three sub-Saharan countries to evaluate the safety and efficacy of MQ as IPTp in HIV-infected women taking daily CTXp and in the context of long lasting insecticide treated nets (LLITNs).

## Methods

### Ethics Statement and Participants' Safety

The study protocol and informed consent forms were reviewed and approved by the Ethics Committees from the Hospital Clínic of Barcelona (Spain), the US CDC, and the local regulatory authorities and National Ethics Review Committees from Kenya, Mozambique, and Tanzania (Table S1). Study participants signed a written informed consent form prior to enrolment. The trial was conducted under the provisions of the Declaration of Helsinki and in accordance with Good Clinical Practices guidelines set up by the WHO and by the International Conference on Harmonization. An independent Data Safety Monitoring Board (DSMB) was created prior to the trial and regularly reviewed and monitored the safety data collected. The trial was registered prior to the enrolment of the first participant in both the ClinicalTrials.gov (NCT00811421) and the Pan African Clinical Trials (2010020001813440) registries.

### Study Area and Population

The study was conducted in three sub-Saharan countries: Tanzania (Makole and Chamwino), Mozambique (Manhiça and Maragra), and Kenya (Siaya). The characteristics of each site are shown in Table S2. Enrolment started in March 2010 and was completed in April 2012; the study follow-up ended in January 2013.

### Study Design

An individually randomized double-blind placebo-controlled trial was conducted to compare the efficacy of three monthly doses of MQ as IPTp with placebo-IPTp in HIV-infected pregnant women receiving daily CTXp in the context of LLITN use. The primary endpoint of the study was the prevalence of peripheral maternal malaria infection (microscopic or submicroscopic) at delivery. Based on previous estimations in the study sites [7],[24], assuming a prevalence of peripheral parasitemia at delivery of 15% with CTX prophylaxis, it was estimated that 453 women per arm were required to detect a decrease of 6.2% or more in the prevalence of peripheral parasitemia in the CTX+IPTp-MQ group (prevalence of 8.7%), with 80% power and 5% two sided significance level. In order to allow 15% losses to follow-up, it was calculated that 535 women/study arm had to be recruited (Texts S1 and S2).

### Enrolment and Interventions

Pregnant women of all gravidities attending the ANC clinic for the first time and who had not received IPTp during their current pregnancy were given the opportunity to be included in the study after providing informed consent. Enrolled women were permanent residents in the area, had a gestational age ≤28 weeks, had a positive HIV-test at recruitment, absence of history of allergy to sulfa drugs or MQ, absence of history of severe renal, hepatic, psychiatric, or neurological disease, and had not received MQ or halofantrine treatment in the preceding four weeks. In Mozambique and Tanzania, HIV-negative women were invited to participate in a randomized controlled trial evaluating the safety and efficacy of MQ IPTp compared to SP IPTp [25]. Gestational age was determined from fundal height measured by bimanual palpation. Following national guidelines in place, HIV status was assessed after voluntary HIV counselling and testing with an HIV rapid test and the positive results confirmed with a second rapid test (details of the rapid tests used are available in Table S2). Haemoglobin (Hb) and the syphilis rapid plasma reagin (RPR) test were assessed as part of routine ANC on fingerprick collected capillary blood, and 5 ml venous blood was taken for CD4+T cell count and viral load determination. Women were recruited regardless of their immunosuppression level (as measured by CD4+T cell count) or whether or not they were already on antiretroviral therapy (ART) for their own health.

The allocation of the participants to the study arms was done centrally by block randomization (block size of 6) stratified by country to receive CTX (Septrin, UCB Pharma, fixed combination 800 mg of trimethroprim and 160 mg of sulfamethoxazole/tablet) plus IPTp-MQ (Lariam, Roche, 250 mg of MQ base/tablet) or CTX plus IPTp-placebo (identical to MQ tablets in shape and colour). The Pharmacy Department of the Hospital Clinic in Barcelona produced and safeguarded the computer-generated randomization list for each recruiting site until unblinding, and carried out the masking, labelling, and packaging of all study interventional drugs. Study number allocation for each participant was concealed in opaque sealed envelopes that were sequentially numbered and opened only after recruitment by study health personnel. Study participants were assigned a unique study number linked to the allocated treatment group. Investigators, laboratory staff, care providers, and study participants were blinded to intervention throughout the study. All participants received a LLITN (PermaNet, Vestergaard Fransen) at enrolment as part of the study intervention.

Following physical examination, recruited women with gestational age ≥13 weeks received the first administration of IPTp (either placebo or MQ) under supervision. The number of tablets administered was calculated based on the participant's body weight at the first IPTp administration at a dosage of 15 mg/kg, assuming that each tablet contained 250 mg of active principle (MQ or placebo). The maximum dosage provided did not exceed 1,500 mg of MQ base. The administration of the study IPTp drugs was supervised and participants were observed for 60 minutes following IPTp administration. Women vomiting within the first 30 minutes were given a second full IPTp dose; those vomiting after 30–60 minutes were given an additional half dose. If the replacement dose was also vomited, study drug dosing was interrupted and routine ANC guidelines followed. The second and third administrations of IPTp-MQ/placebo were given at least one month apart. All women also received study CTX tablets on a monthly basis for daily prophylaxis.

### Follow-up

Drug tolerability was assessed immediately and two days after intake by home visits of field workers. Adherence to CTXp (measured by self-reported use and by counting the remaining number of tablets) and to LLITNs (assessed by self-reported use the preceding night) was evaluated at every monthly ANC visit, as well as during all unscheduled visits. Women were encouraged to attend the study health facility whenever they had any health complaint. Health care was free of charge and in general there was little availability of antimalarial drugs over the counter at all sites. A health facility-based passive surveillance system was established at each site to capture unscheduled visits of study participants during study follow-up. At each unscheduled visit, a standardized questionnaire was completed documenting signs and symptoms. Blood smears were prepared for malaria parasite examination and Hb was measured if there were current or reported symptoms and/or signs suggestive of malaria. Clinical malaria episodes were treated with oral quinine (first trimester) or artemether-lumefantrine (subsequent trimesters) for uncomplicated malaria; parenteral quinine was used for treatment of severe malaria. Solicited and unsolicited adverse events (AEs) were assessed. The former was done by directed questioning of malaria related signs and symptoms during unscheduled visits, whereas the latter were assessed through open questioning during scheduled visits. HIV/AIDS management of study participants was provided by the local government health services according to national guidelines [26]–[28]. Administration of antiretroviral (ARV) drugs for prevention of mother to child transmission of HIV (PMTCT) or ART was registered in the study concomitant medication forms.

At delivery, a sample from the mother's peripheral blood was collected for Hb, CD4+T cell count, HIV viral load, and malaria infection evaluation; cord blood and placental samples (biopsy and impression smears) were also taken, as well as blood onto filter paper for qPCR determinations. Newborns were weighed (including stillbirths) and measured and their gestational age at birth assessed using the Ballard's score [29]. Babies' weights not captured at birth were estimated from weights obtained in the first week of life using a regression model [30]. Six weeks after the end of pregnancy, a capillary blood sample from the mother was collected for malaria parasite determination. Infants born to study participants were followed until two months after birth to assess survival and general morbidity. Following national guidelines for PMTCT of HIV, a capillary blood sample was collected from the infant at six weeks of age onto filter paper for HIV PCR analysis.

### Laboratory Methods

Maternal HIV and syphilis serostatus were assessed at each site according to local standard procedures using rapid diagnostic tests (Table S2). CD4+T cell count was determined by flow cytometry after staining of whole blood with CD3, CD8, and CD4 fluorochrometolabelled antibodies and acquisition using FACSCalibur (BD Biosciences) and TruCOUNT tubes (Becton Dickinson). HIV viral load was determined from plasma cryopreserved at −80°C using the COBAS AMPLICOR or AmpliPrep (Roche Diagnostics) devices; these assays have a lower detection limit ranging from 50 to 400 copies per millilitre. Hb levels were determined using mobile devices (HemoCue [www.eurotrol.com] and Hemocontrol [www.ekfdiagnostics.com]) on capillary blood samples. *Plasmodium falciparum* parasites were identified by microscopy on Giemsa-stained blood films according to standard, quality-controlled procedures [31]–[33]. *P. falciparum*-specific real time quantitative PCR (RT-qPCR) was performed on maternal peripheral and placental samples, with the inclusion in all reactions of a negative control with no template DNA [34]. Tissue samples were collected from the maternal side of the placenta and fixed with 10% neutral buffered formalin. Biopsies were processed, stained, and examined following standard procedures [35]. Impression smears from the placenta were stained with Giemsa's staining and read following a standardized protocol [36].

### Data Management, Statistical Methods, and Definitions

The quality of the data recorded in the study source documents and case report forms (CRFs) were monitored regularly following the principles of Good Clinical Practices by the trials' clinical monitor before their shipment to the centralized database. Data were double-entered using the OpenClinica Enterprise software for clinical data management (www.openclinica.com). The main analysis was done on the Intention-to-Treat (ITT) cohort that included all recruited women that met the inclusion criteria and had data on the specific outcomes, and on the According to Protocol (ATP) cohort that included all women who had received three doses of IPTp according to the pre-specified schedule, and have results available on peripheral parasitemia at delivery. The safety cohort was defined as all recruited women who had received at least one dose of IPTp and whose data for analysis were available. The analysis of safety and tolerability was made on the Safety cohort. The ITT analyses were adjusted by country. The analyses in the ATP cohort were adjusted by country only and by baseline covariates (country, gestational age, gravidity, anemia, literacy, middle upper arm circumference [MUAC], and CD4+T cell count). The proportion of women with asexual *P. falciparum* parasitemia (either microscopic or submicroscopic) was compared between groups using a modified binomial regression [37]. The statistical analysis plan is available in Text S3. Findings of all study outcomes listed in the protocol (Text S1) are reported in the results section and the tables.

Peripheral malaria infection at delivery (primary study endpoint) was defined as the presence of asexual *P. falciparum* parasites of any density in a blood smear or filter paper (detected either by optical microscopy or PCR, respectively). Heterogeneity between countries for the primary endpoint was evaluated using a Wald test. A clinical malaria episode was defined as presence of *P. falciparum* parasites in a blood smear plus any sign or symptom suggestive of malaria including: current fever (axillary temperature ≥37.5°C) or history of fever in the last 24 hours, and/or pallor, and/or arthromyalgias and/or headache, and/or history of convulsions [38]. The incidence of all-cause hospital admissions and all-cause outpatient attendance was analyzed using negative binomial regression. The same statistical method was used for the exploratory analysis of non-obstetric causes of hospital admission. The incidence of all clinical malaria episodes was compared between groups also using a negative binomial regression allowing for interdependence between episodes within the same subject, excluding from the time at risk the 28 days after the end of treatment for a malaria episode. Detection of parasites in blood samples by RT-qPCR where the corresponding blood smear was read as negative was defined as submicroscopic *P. falciparum* infection.

Placental infection was defined as the presence of parasites with or without pigment detected by histological examination, impression smear [35] or PCR [34]. Maternal anemia was defined as a Hb level <11 g/dl and severe anemia as Hb<7 g/dl, while fetal anemia was defined as a cord blood Hb level <12.5 g/dl. CD4+T cell count was categorized as ≤350 or >350 cells/µl. Maternal viral load at delivery was analyzed in the logarithmic scale using censored regression (Tobit regression) including as censored values those that were lower than 400 copies/ml [39],[40]. Coefficients of the regression were transformed back to original scale and presented as proportional difference between groups. Adherence with CTXp was evaluated independently in each country using the Wilcoxon Rank sum test. Ordered polytomous logistic regression on the tertiles of CTXp adherence was used to evaluate the adherence by intervention group adjusting by country (and other baseline covariates in the ATP analysis). Participant's adherence with PMTCT or ART was estimated from self-reporting and using the concomitant medication registry, and classified as follows: complete, ARVs received antenatally, intrapartum, and postpartum, in accordance to national guidelines [26]–[28]; incomplete, if ARVs received partially only; or nothing, if no ARVs were registered or reported. The proportion of HIV-infected infants was compared between groups using a similar methodology to that used for the primary outcome [34]. As an exploratory analysis to evaluate possible confounders on the rates of MTCT transmission of HIV, a univariate analysis for selected variables (namely PMTCT adherence, type of delivery [c-section *versus* vaginal], health facility delivery, presence of moderate vomiting as AE, drug-related vomiting, maternal hospital admissions, clinical malaria during pregnancy, placental infection, RPR test result at baseline, and viral load at delivery) was performed and only those statistically significant variables (*p*<0.05) were included in the multivariate model.

Immediate tolerability to study drugs was assessed as observed vomiting within one hour of drug administration. An AE was defined as any untoward medical occurrence in a study participant, to whom the study drug was administered, including occurrences, which were not necessarily caused or related to that drug. Serious adverse events (SAEs) were defined as an AE that meets any of the following criteria: (a) results in death, (b) is life-threatening, (c) requires hospitalization (or prolongation of existing hospitalization), (d) results in disability/incapacity, (e) is a congenital anomaly, or (f) any event of special interest (including cutaneous and neuropsychiatric events, miscarriages, and stillbirths of women not admitted to hospital) [41]. The proportions of women with an AE or a SAE were presented by treatment group with 95% exact confidence intervals. For safety and tolerability outcomes it was considered that there was no evidence of significant difference between treatment groups if the 95% confidence intervals overlapped. Data analysis was performed using Stata statistical software version 13 (Stata Corp.).

## Results

**Figure 1:**
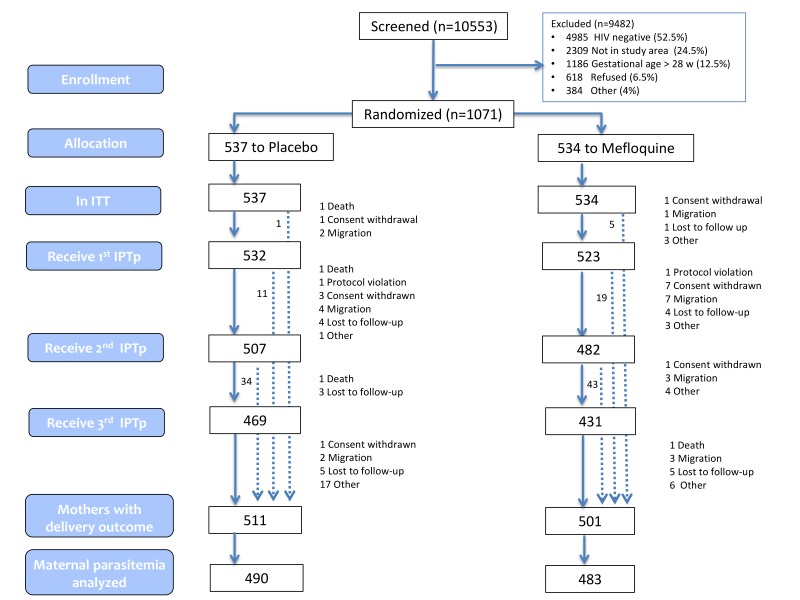
Trial profile (ITT cohort).

Figures 1 and S1 show the trial profiles for the ITT and the ATP cohorts. Overall, 1,071 pregnant women were randomized to receive IPTp (537 were allocated to control and 534 to MQ). Tanzania contributed with 45 participants only because of competitive recruitment with another study in the area. The main reasons for non-enrolment into the trial were baseline HIV test negative (52.5%), no permanent residence in the study area (24.5%), and gestational age >28 weeks (12.5%). The overall refusal rate for trial participation was 6.5%. Baseline characteristics were similar between treatment groups (Table 1). Mean gestational age was 21 weeks (standard deviation [SD] 7) at the first IPTp dose, 26 weeks (SD 6) at the second IPTp dose, and 29 weeks (SD 6) at the third IPTp dose. Median time between the first and the second IPTp dose was 32 days (IQR 2), 31 days (IQR 2) between the second and the third, and 62 days (IQR 49) between the last dose and delivery. The second IPTp dose was not given to 4.7% and 7.8% of women who had received the first dose of placebo and MQ, respectively. The third dose was not given to 7.5% and 10.6% of the women who had received the second dose of placebo and MQ, respectively. The reasons for not receiving IPTp doses are shown in the trial profile (Figure 1). Overall, adherence to CTXp was high with over 66% of pregnant women taking 87% or more of the expected dosages (75% took 80% or more) and with no significant differences between groups (Table 2). Compared with Kenya, the odds of being in a higher category of adherence to CTXp was lower in Mozambique (odds ratio [OR] 0.31, [95% CI 0.24–0.39]; *p*<0.001) or in Tanzania (OR, 0.16, [95% CI 0.09–0.30]; *p*<0001). The OR of being in a higher category of adherence to CTXp in the MQ group compared with the placebo group was 0.86 ([95% CI 0.68–1.09]; *p* = 0.203) and there was no interaction between country and intervention group (Chi-square 0.33 with 2 degrees of freedom [df]; *p* = 0.846).

**Table 1:** Baseline characteristics.

Variables		Control	MQ
Participants	*N*	537	534
Country^a^	Kenya	234 (44)	231 (43)
	Mozambique	280 (52)	281 (53)
	Tanzania	23 (4)	22 (4)
Age (years)^b^		26.6 (5.4) [536]	26.8 (5.8) [533]
Gravidity (categories)^a^	Primigravidae	51 (9)	57 (11)
	1–3 previous pregnancies	363 (68)	341 (64)
	4 or more pregnancies	122 (23)	136 (25)
Weight (kg)^b^		59.8 (8.1) [537]	60.2 (8.8) [534]
Height (cm)^b^		161.1 (7.4) [537]	161.3 (8.3) [532]
MUAC (cm)^b^		26.9 (2.6) [534]	26.9 (3.1) [528]
Gestational age (weeks)^c^		21.0 (7.0) [537]	21.0 (8.0) [534]
Gestational age in categories^a^	First trimester	70 (13)	62 (12)
	Second trimester	340 (63)	343 (64)
	Third trimester	127 (24)	129 (24)
Literate (can read and/or write)^a^	No	111 (21)	91 (17)
	Yes	426 (79)	443 (83)
Syphilis test^a^	Positive	25 (5)	29 (5)
	Negative	510 (95)	501 (94)
Hb (g/dl)^b^		10.2 (1.7) [535]	10.1 (1.8) [533]
Overall anemia (Hb<11 g/dl) at baseline^a^	No	191 (36)	169 (32)
	Yes	346 (64)	365 (68)
Viral load categories (copies/ml)^a^	Undetectable	109 (20)	120 (22)
	400–999	130 (24)	122 (23)
	1,000–9,999	167 (31)	169 (32)
	>9,999	101 (19)	91 (17)
CD4 categories (c/µl)^a^	≤350	206 (38)	197 (37)
	>350	310 (58)	316 (59)
Start ART after recruitment^a^	No	72 (14)	90 (17)
	Yes	193 (37)	175 (33)
	ART indicated at baseline	263 (50)	262 (50)
On ART at baseline		119 (22)	130 (24)

ITT cohort.

a *n* (column percentage).

b Arithmetic mean (SD) [n].

c Median (IQR) [*n*].

MUAC, middle upper arm circumference.

**Table 2:** Adherence to cotrimoxazole categorized in tertiles.

CTX Adherence	Control		MQ		OR^a^ (95%CI)	*p*-Value
	*n*	Percent	*n*	Percent		
**ITT**					0.87 (0.89–1.09)	0.217
Tertile 1 (0%<87%)	161	31.5	176	35.2		
Tertile 2 (87%<100%)	130	25.4	124	24.8		
Tertile 3 (100%)	220	43.1	200	40.0		
**ATP**					0.99 (0.77–1.27)	0.952
Tertile 1 (0%<87%)	133	30.1	127	30.5		
Tertile 2 (87%<100%)	119	26.9	110	26.4		
Tertile 3 (100%)	190	43.0	179	43.0		

a OR of being in a given category or superior. ITT adjusted by country. ATP analysis adjusted by baseline variables (country, literacy, gestational age, gravidity, anemia, middle upper arm circumference, and CD4). Interaction MQ×country, *p*-value = 0.842 for ITT, *p*-value = 0.771 for ATP cohort.

### Primary Endpoint

A total of 973 peripheral blood samples from women at delivery were collected (490 from control and 483 from MQ group). The prevalence of peripheral maternal malaria infection at delivery was significantly lower in women receiving IPTp-MQ compared to those who received IPTp-placebo (7.6% in the control group and 3.5% in the MQ group; risk ratio (RR) 0.47 [95% CI 0.27–0.82]; *p* = 0.008) (no interaction between country and treatment group was found [Chi-square 4.27 with 2 df; *p* = 0.11]). Similar results were found in the ATP analysis (Table 3).

**Table 3:** Malaria-related outcomes at delivery.

Outcomes	Control		MQ		RR or Difference	95% CI	*p*-Value
	*n*/*N*	Percent	*n*/*N*	Percent			
Maternal parasitemia (smear or PCR)							
ITT [*N* = 973]	37/490	7.6	17/483	3.5	0.47^a^	(0.27–0.82)	0.008
ATP [*N* = 859]	31/443	7.0	16/416	3.8	0.54^a^	(0.30–0.97)	0.038
Placental infection (histology, smear, or PCR)							
ITT [*N* = 911]	34/462	7.4	17/449	3.8	0.52^a^	(0.29–0.90)	0.021
ATP [*N* = 808]	30/418	7.2	16/390	4.1	0.51^a^	(0.30–0.89)	0.017
Maternal anemia (Hb<11 g/dl)							
ITT [*N* = 963]	187/484	38.6	190/479	39.7	1.02^a^	(0.88–1.19)	0.758
ATP [*N* = 849]	155/436	35.6	161/413	39.0	1.11^a^	(0.94–1.31)	0.232
Severe maternal anemia (Hb<7 g/dl)							
ITT [*N* = 963]	12/484	2.5	11/479	2.3	0.93^a^	(0.41–2.08)	0.857
ATP [*N* = 849]	10/436	2.3	10/413	2.4	0.89^a^	(0.35–2.27)	0.814
Maternal Hb, mean (SD) [n]							
ITT [*N* = 963]	11.3 (2.2) [484]		11.2 (2.1) [479]		−0.03^b^	(−0.28 to 0.22)	0.826
ATP [*N* = 849]	11.4 (2.2) [436]		11.3 (2.1) [413]		−0.09^b^	(−0.35 to 0.18)	0.521
Low birth weight (<2,500 g)							
ITT [*N* = 975]	46/486	9.5	61/489	12.5	1.32^a^	(0.90–1.95)	0.157
ATP [*N* = 865]	35/441	7.9	48/424	11.3	1.39^a^	(0.89–2.17)	0.147
Birth weight, mean (SD) [n]							
ITT [*N* = 975]	3,059.3 (575.5) [486]		3,036.3 (570.6) [489]		−22.50^b^	(−98.31 to 53.32)	0.561
ATP [*N* = 865]	3,100.4 (529.9) [441]		3,055.6 (540.4) [424]		−38.84^b^	(−114.01 to 36.33)	0.312
Gestational age at birth (weeks), mean (SD) [n]^c^							
ITT [*N* = 436]	38.8 (1.1) [230]		38.7 (1.3) [236]		−0.10^b^	(−0.32 to 0.13)	0.405
ATP [402]	38.9 (1.1) [205]		38.8 (1.2) [197]		−0.07^b^	(−0.32 to 0.17	0.551
Cord blood parasitemia (smear)							
ITT [*N* = 933]	3/462	0.6	1/471	0.2	0.33^a^	(0.03–3.13)	0.334
ATP [*N* = 836]	3/424	0.7	1/412	0.2	0.24^a^	(0.04–1.49)	0.126
Cord blood anemia (Hb<12.5 g/dl)							
ITT [*N* = 930]	67/459	14.6	80/471	17.0	1.17^a^	(0.87–1.58)	0.303
ATP [*N* = 835]	63/424	14.9	73/411	17.8	1.20^a^	(0.88–1.64)	0.257
Maternal parasitemia one month post- delivery (smear)							
ITT [*N* = 836]	7/423	1.7	8/413	1.9	1.20^a^	(0.44–3.26)	0.721
ATP [*N* = 732]	6/377	1.6	8/355	2.3	1.45^a^	(0.55–3.87)	0.454

a RR.

b Mean difference.

c Assessed by the Ballard score (excluding incomplete data). ITT analysis adjusted by country. ATP analysis adjusted by baseline variables (country, literacy, gestational age, gravidity, anemia, middle upper arm circumference, and CD4).

### Secondary Endpoints

The frequency of placental infection was significantly lower in the MQ group (3.8%) compared to the control group (7.4%) (RR, 0.52, [95% CI 0.29–090]; *p* = 0.021). There were no significant differences between groups in the prevalence of maternal and fetal anemia, prevalence of low birth weight, or in the prevalence of fetal and maternal parasitemia one month after delivery (Table 3). At delivery, in the ITT analysis the proportion of women with low maternal CD4+T cell count and the distribution of viral load were similar between groups. In the ATP analysis the viral load distribution in the MQ group was 1.61 times (95% CI 1.00–2.59) higher than in the control group (*p* = 0.048) (Table 4).

**Table 4:** HIV-related outcomes at delivery.

Variable	Control		MQ		RR or Difference	95% CI	*p*-Value
	*n*	Percent	*n*	Percent			
Maternal CD4 counts >350 c/µl							
ITT [*N* = 873]	244	55.8	235	53.9	0.97^a^	(0.86–1.09)	0.561
ATP [*N* = 768]	216	55.0	199	53.1	0.93^a^	(0.82–1.04)	0.212
Viral load categories (copies/ml)							
ITT					1.40^b^	(0.90–2.18)	0.134
Undetectable	179	33.3	161	30.1			
400–999	33	6.1	19	3.6			
1,000–9,999	215	40.0	236	44.2			
>9,999	40	7.4	45	8.4			
No data	70	13.0	73	13.7			
ATP					1.61^b^	(1.00–2.59)	0.048
Undetectable	168	37.9	142	34.1			
400–999	32	7.2	17	4.1			
1,000–9,999	188	42.4	198	47.6			
>9,999	33	7.4	41	9.9			
No data	22	5.0	18	4.3			

a RR.

b Proportional difference. *p*-Value from censored regression (Tobit) using Wald test. Treatment comparison adjusted by country. Viral loads lower than 400 are considered as censored in the regression analysis. ITT analysis adjusted by country. ATP analysis adjusted by baseline variables (country, literacy, gestational age, gravidity, anemia, middle upper arm circumference, and CD4).

The incidence of hospital admissions that were either all-cause or only due to non-obstetric causes was lower in the women receiving MQ compared to those receiving placebo (RR, 0.65, [95% CI 0.41–1.03]; *p* = 0.065) and (RR, 0.59, [95% CI 0.37–0.95]; *p* = 0.031), respectively. The incidences of clinical malaria and all-cause outpatient attendance during the study follow-up tended to be lower in women receiving MQ than in those receiving placebo, although these differences were not statistically significant (RR, 0.52, [95% CI 0.22–1.21]; *p* = 0.128; and RR, 0.86 [95% CI 0.72–1.03]; *p* = 0.098, respectively) (with similar results in the ATP analysis) (Table 5; Figure S2). Overall, no differences were found between the ATP unadjusted and the ATP adjusted analyses.

**Table 5:** Incidence of clinical malaria, outpatient visits, and hospital admissions.

Outcome	Control		MQ		Relative Rate	95% CI	*p*-Value
	*n*/PYAR^a^	Incidence	*n*/PYAR^a^	Incidence			
Clinical malaria							
ITT	16/189.1	0.09	8/182.2	0.04	0.52	(0.22–1.21)	0.128
ATP	15/169.3	0.09	7/158.2	0.04	0.50	(0.20–1.23)	0.132
Outpatient visits							
ITT	401/190.2	2.11	332/182.8	1.82	0.86	(0.72–1.03)	0.098
ATP	361/170.4	2.12	302/158.7	1.90	0.90	(0.75–1.08)	0.271
All-cause hospital admissions							
ITT	68/190.2	0.36	41/182.8	0.22	0.65	(0.41–1.03)	0.065
ATP	52/170.4	0.31	26/158.7	0.16	0.53	(0.31–0.90)	0.019
Non-obstetric hospital admissions							
ITT	67/190.2	0.35	37/182.8	0.20	0.59	(0.37–0.95)	0.031
ATP	51/170.4	0.30	22/158.7	0.14	0.47	(0.27–0.82)	0.007

a Episodes per person/year. ITT analysis adjusted by country. ATP analysis adjusted by baseline variables (country, literacy, gestational age, gravidity, anemia, middle upper arm circumference, and CD4).

PYAR, person/year.

The overall reported use of LLITNs at delivery was of 93% and of 98% one month after the end of pregnancy, with no difference between study groups.

### Safety

There were no differences in the prevalence of miscarriages, stillbirths, premature births, and congenital malformations between groups (Table 6). The number of SAE, including maternal and neonatal deaths, was also similar between study arms. None of the reported SAEs were considered to be drug-related by the clinical investigator assessing the participant.

**Table 6:** Adverse pregnancy outcomes and serious adverse events by study arm (safety cohort).

Adverse Event	Control			MQ			*p*-Value
	*n*	Percent	95% CI	*n*	Percent	95% CI	
Adverse pregnancy outcomes							
Miscarriages^a^	6	1.1	(0.41–2.44)	2	0.4	(0.05–1.37)	0.287
Stillbirths^b^	22	4.1	(2.61–6.19)	18	3.4	(2.05–5.38)	0.630
Congenital malformations	8	1.6	(0.67–3.04)	5	1.0	(0.32–2.30)	0.579
Prematurity^c^^,^ d	9	3.2	(1.45–5.91)	14	4.9	(2.72–8.13)	0.297
SAEs							
Any SAE	74	13.9	(11.08–17.14)	48	9.2	(6.84–11.98)	0.021
SAEs related to medication	0	0.0	(0.00–0.69)	0	0.0	(0.00–0.70)	—
Maternal deaths	4	0.8	(0.21–1.91)	2	0.4	(0.05–1.37)	0.687
Neonatal deaths	10	2.0	(0.94–3.56)	13	2.6	(1.37–4.34)	0.535
Perinatal deaths^e^	30	5.8	(3.96–8.21)	30	5.9	(4.03–8.34)	0.963

a Miscarriage: termination of pregnancy and expulsion of an embryo or of a foetus prior to 20 complete weeks of gestation (as estimated by measurement of fundal height) and/or a birth weight less than 500 g.

b Stillbirth: foetal death that occurs after 20 complete weeks of gestation.

c Prematurity: birth before the beginning of the 37th week (assessed by the Ballard score).

d Excluding incomplete data on Ballard score.

e Perinatal death: foetal death that occurs during late pregnancy (at ≥28 completed weeks of gestation), childbirth and neonatal deaths within the first seven days of life.

### Tolerability

The overall immediate tolerability of IPTp was poorer in the MQ group (25 episodes of vomiting in the first hour following IPTp administration) as compared to placebo (one episode of vomiting) (Table 7). The most frequently reported AEs following the first IPTp administration were dizziness, vomiting, nausea, and headache in both groups. These frequencies were significantly higher in the MQ group as compared to the control group and were reduced in the subsequent second and third IPTp administrations (Table 8). The majority of the reported related AEs were mild (Table 9).

**Table 7:** Immediate medication tolerability (safety cohort).

Adverse Event	Control			MQ		
	*n*	Percent	95% CI	*n*	Percent	95% CI
1st IPTp administration						
Vomiting within 30 min	0	0.0	(0.00–0.69)	6	1.2	(0.42–2.48)
Vomiting within 60 min	0	0.0	(0.00–0.69)	6	1.2	(0.42–2.48)
Vomiting replacement dose	0	0.0	(0.00–0.69)	3	0.6	(0.12–1.67)
2nd IPTp administration						
Vomiting within 30 min	0	0.0	(0.00–0.73)	6	1.2	(0.46–2.69)
Vomiting within 60 min	0	0.0	(0.00–0.73)	4	0.8	(0.23–2.11)
Vomiting replacement dose	0	0.0	(0.00–0.73)	4	0.8	(0.23–2.11)
3rd IPTp administration						
Vomiting within 30 min	1	0.2	(0.01–1.18)	2	0.5	(0.06–1.67)
Vomiting within 60 min	0	0.0	(0.00–0.79)	1	0.2	(0.01–1.29)
Vomiting replacement dose	0	0.0	(0.00–0.79)	0	0.0	(0.00–0.85)

**Table 8:** Most frequent medication related adverse events (safety cohort).

Adverse Event	Control			MQ		
	*n*	Percent	95% CI	*n*	Percent	95% CI
1st IPTp administration						
Dizziness	40	7.5	(5.43–10.10)	155	29.6	(25.75–33.75)
Vomiting	16	3.0	(1.73–4.84)	125	23.9	(20.31–27.79)
Nausea	21	4.0	(2.46–5.97)	54	10.3	(7.85–13.26)
Headache	40	7.5	(5.43–10.10)	38	7.3	(5.19–9.84)
2nd IPTp administration						
Dizziness	17	3.4	(1.97–5.31)	86	17.8	(14.53–21.56)
Vomiting	11	2.2	(1.09–3.85)	76	15.8	(12.63–19.33)
Nausea	8	1.6	(0.68–3.09)	29	6.0	(4.07–8.53)
Headache	23	4.5	(2.90–6.73)	29	6.0	(4.07–8.53)
3rd IPTp administration						
Dizziness	9	1.9	(0.88–3.61)	42	9.7	(7.11–12.94)
Vomiting	12	2.6	(1.33–4.43)	36	8.4	(5.92–11.38)
Nausea	3	0.6	(0.13–1.86)	24	5.6	(3.60–8.17)
Headache	24	5.1	(3.31–7.52)	30	7.0	(4.75–9.79)

**Table 9:** Severity of reported vomiting and dizziness by treatment group.

Severity Grade	Control		MQ	
	*n*	Percent	*n*	Percent
**Vomiting related to medication**				
Mild^a^	28	71.8	196	80.3
Moderate^b^	10	25.6	42	17.2
Severe^c^	1	2.6	6	2.5
**Dizziness related to medication**				
Mild^a^	55	78.6	217	75.6
Moderate^b^	15	21.4	61	21.3
Severe^c^	0	0.0	9	3.1

a Mild: awareness of sign or symptom, but easily tolerated.

b Moderate: discomfort enough to cause interference with usual activity.

c Severe: incapacitating with inability to work or perform usual activity or patients at risk of death at the time of the event.

### Mother to Child Transmission of HIV

Given the observed difference between the treatment groups in viral load at delivery, a more detailed analysis of the mother to child transmission (MTCT) of HIV at 6 weeks of age (median 5.9 weeks, IQR 1.7) was performed. The proportion of infants with an HIV DNA positive PCR was higher in those born to women who had received MQ compared to those who had received placebo (RR, 1.95, [95% CI 1.14–3.33]; *p* = 0.015; with similar results in the ATP analysis) (Table 10). There was no difference in the proportion of mother-infant pairs with complete PMTCT of HIV or ART regimen by treatment group based on registry examination (233/435 [54%] in the control group and 249/420 [59%] in the MQ group). Table 11 shows the concomitant ARV medication received by treatment group. There was no difference in the mode of delivery by group (6% underwent a C-section in the control group and 5% in the MQ group), nor in the proportion of hospital deliveries (86% in both intervention groups). Reported vomiting (either related or not, or severe) was not associated with increased viral load (*p* = 0.81), nor with the risk of MTCT of HIV (*p* = 0.655). In contrast, clinical malaria during pregnancy was found to be associated with an increased risk of MTCT of HIV. Higher viral loads at delivery were also associated with increased risk of MTCT of HIV (Table 12).

**Table 10:** Mother to child transmission of HIV by treatment group.

Infant HIV PCR Results^a^	Control		MQ		RR (95% CI)	*p*-Value
	*n*	Percent	*n*	Percent		
**ITT** [*N* = 855]						
Positive	19	4.4	36	8.6	1.95	0.015
Negative	416	95.6	384	91.4	(1.14–3.33)	
**ATP** [*N* = 754]						
Positive	15	3.8	29	8.0	2.10	0.016
Negative	378	96.2	332	92.0	(1.15–3.84)	

a Median age 5.9 weeks (IQR 1.7). ITT analysis adjusted by country. ATP analysis adjusted by baseline variables: country, literacy, gestational age, gravidity, anemia, middle upper arm circumference and CD4 counts at baseline. Interaction MQ×country = *p*-value 0.660 for ITT cohort, and 0.872 for ATP cohort.

**Table 11:** Concomitant antiretroviral medication by treatment group (ITT cohort).

Drugs	*n* (%) Control	*n* (%) MQ
Zidovudine	391 (72.8)	368 (68.9)
Nevirapine	386 (71.9)	375 (70.2)
Lamivudine	347 (64.6)	345 (64.6)
Stavudine	64 (11.9)	70 (13.1)
Tenofovir Disproxil Fumarate	17 (3.2)	19 (3.6)
Efavirenz	4 (0.7)	9 (1.7)
Lopinavir	0 (0.0)	3 (0.6)
Ritonavir	0 (0.0)	3 (0.6)
Abacavir	2 (0.4)	1 (0.2)
Emtricitabine	0 (0.0)	1 (0.2)

**Table 12:** Multivariate analysis of risk factors for mother to child transmission of HIV.

Variable	ITT			ATP		
	RR	CI 95%	*p*-Value	RR	CI 95%	*p*-Value
Treatment						
MQ vs control	2.05	1.16–3.63	0.014	2.17	1.12–4.19	0.021
Viral load at delivery (copies/ml)			0.022			0.100
400–999 vs <400	4.80	1.38–16.65		3.32	0.88–12.50	
1,000–9,999 vs <400	3.59	1.39–9.29		3.75	1.43–9.87	
>9,999 vs <400	5.82	2.01–16.84		3.62	1.14–11.51	
No data vs <400	2.78	0.80–9.74		1.22	0.16–9.20	
Clinical malaria episodes in pregnancy^a^	3.05	1.35–6.92	0.008	4.76	2.01–11.24	<0.001
Maternal adherence to PMTCT or ART guidelines			0.010			0.042
Incomplete^b^ vs complete^c^	1.94	1.06–3.57		1.96	0.98–3.92	
Nothing^d^ vs complete	2.86	1.43–5.74		3.01	1.22–7.37	

Median age of infants was 5.9 weeks (IQR 1.7) at the time of the HIV PCR test. Analysis adjusted by baseline variables: country, literacy, gestational age, gravidity, anemia, middle upper arm circumference, CD4 counts and viral load. ITT analysis adjusted by country. ATP analysis adjusted by baseline variables: country, literacy, gestational age, gravidity, anemia, middle upper arm circumference, CD4 counts, and viral load.

a At least one episode of clinical malaria during study follow-up in pregnancy.

b Incomplete: received partially PMTCT (either antenatal, intrapartum, or postpartum) or ART.

c Complete: received PMTCT (antenatal, intrapartum, and postpartum) or ART according to national guidelines.

d The mother did not receive either PMTCT or ART.

## Discussion

This placebo-controlled trial of IPTp with MQ (15 mg/kg dose three times at least one month apart) in HIV-infected pregnant women in the context of daily CTXp and LLITN use found significant reductions in the risk of important parameters of malaria infection in pregnancy such as maternal parasitemia at delivery and placental infection. In addition, IPTp-MQ was associated with reduction in the incidence of non-obstetric hospital admissions while the incidence of all-cause out-patient visits, all-cause hospital admissions, and clinical malaria during pregnancy also tended to be lower in women receiving IPTp-MQ. The safety profile in terms of SAEs and adverse pregnancy outcomes was similar between the two study groups, and there were no reported SAEs related to the medication. On the other hand, tolerability of MQ was poor compared to the placebo group especially for the AEs that have been commonly reported with this drug such as dizziness and vomiting. MQ IPTp was also associated with an increased risk of MTCT of HIV.

Reduced frequency of placental malaria in MQ recipients was also reported in an open-label trial comparing CTXp and IPTp-MQ plus CTXp in 292 HIV-infected Beninese women [23]. In the present study, IPTp-MQ was associated with a reduction in the incidence of morbidity outcomes both overall and malaria-related, and this finding was consistent with a reduction in the incidence of non-obstetric admissions, which were mainly caused by infectious causes. HIV-infected pregnant women have an increased risk of suffering and dying from infectious diseases [42]–[45]. On the other hand, it is well known that malaria infection causes immunosuppression, increasing the risk and severity of other infections [3],[46]. This increased risk of immunosuppression is supported by the findings of several studies testing malaria preventive interventions in children that have shown a reduction in overall (not just malaria-specific) morbidity and mortality due to malaria preventive interventions in children [47]. Thus, it is possible that the decreased risk of malaria infection in women who took MQ had an impact in reducing the incidence and/or severity of other infections. It may also be speculated that women receiving MQ were less likely to go to the health facility because they were more likely to suffer AEs (mainly dizziness and vomiting). Although we do not have information to refute this possibility, it seems unlikely that these AEs that were generally mild and transient would have prevented severely ill women from attending the health facility.

The poor tolerability of MQ compared to placebo found in this study has been reported previously [48] and in a recent trial of IPTp with the same MQ dose among HIV-negative pregnant women [25]. This finding is in contrast with the results from a double-blind placebo-controlled trial of MQ prophylaxis using a different regimen and lower dose (250 mg/weekly for one month followed by 125 mg/weekly until delivery) among pregnant women from Thailand, where similar rates of adverse effects were reported among the placebo and the MQ group [48]. In the present study, MQ tolerability appeared to improve with each subsequent dose of MQ. The reduced frequency of reported AEs with repeated number of administrations has been observed in other studies of MQ among pregnant women but also in non-pregnant women travelling in malaria endemic regions [21],[49]–[51]. This study confirmed the safety of MQ with regard to stillbirths, which has also been observed among HIV-negative women [21],[25].

In an exploratory analysis, the study also found that although similar at baseline, HIV viral loads at delivery were significantly higher in the women who received MQ than in those who received placebo, and the proportion of infants who were perinatally infected with HIV was also increased if their mothers received MQ. The effect of MQ on HIV viral loads at delivery and on MTCT was not assessed in the trial evaluating MQ for IPTp in HIV-infected Beninese pregnant women, but the MTCT frequency was too low (less than 1%), given the sample size of the study population to carry out this analysis [23]. The unexpected finding of increased viral loads in women in the MQ group led us to conduct an exploratory analysis of the association of MTCT of HIV by group and of the factors associated with it. The reasons for these associations are yet to be elucidated, though several hypotheses could be postulated. First, the association could be related to a poorer adherence with PMTCT and ART regimens in the MQ group. However, though adherence with ARVs was associated with the risk of MTCT of HIV, no differences were found between groups. Second, despite an adequate adherence with ARVs, the increased frequency of vomiting in the MQ group could have contributed to the association with a higher frequency of MTCT due to a reduced absorption of the ARVs. Nevertheless, MQ-related vomiting was generally mild and transient and thus unlikely to have significantly affected the absorption of drugs taken throughout pregnancy. Third, an as yet undefined pharmacokinetic interaction between MQ and the ARVs used for PMTCT and ART (see Table 10 for the list of ARVs) could have resulted in lower efficacy to these drugs [52]. Interestingly, despite the geographical overlap of malaria and HIV in many countries and the challenging management of the co-infection, information regarding pharmacokinetic drug interactions of antimalarial and ARVs is mainly limited to artemisinin derivatives and in general indicates little effect of the former on the pharmacokinetics of ARVs [53]–[57]. On the other hand, an attenuation of immunization has been described when MQ was administered concurrently with the live attenuated Salmonella typhi oral vaccine indicating an immunemodulator effect of MQ [58],[59], which is supported by the use of MQ in the treatment of progressive multifocal leukoencephalopathy (a severe demyelinating disease that occurs in immunosuppressed individuals) with favourable outcomes [60]–[62]. Thus, MQ might have played an immunemodulator role in study participants, resulting in an increase in viral load and increased frequency of MTCT of HIV. The main limitation of the findings on the association of MQ with MTCT of HIV is the exploratory nature of this analysis. However, these results suggest further studies on the pharmacological interactions between antimalarials and ARV drugs are needed given the enormous public health relevance of both infections. Finally, this unexpected finding indicates that assessment of MTCT of HIV should be included in studies evaluating the efficacy and safety of antimalarials in HIV-infected pregnant women.

An association of presumptive clinical malaria with increased risk of MTCT of HIV has been reported [63]. In the current study, it seemed contradictory that both clinical malaria and MQ were associated with an increased frequency of MTCT of HIV, since MQ was also related with decreased malaria risk. Although these associations could be due to chance, in the multivariate model the effects of clinical malaria and MQ increasing the frequency of perinatal MTCT of HIV were independent. The independence of the two effects is supported by the fact that in both the univariate and multivariate models the magnitude of each association with MTCT of HIV was similar, and the interaction was not significant. It could be hypothesized that this observed increased frequency of MTCT in association with clinical malaria is explained not by an effect of malaria itself but rather by pharmacokinetic interactions between the artemisinin derivatives, which the women received to treat malaria episodes, and the ARVs taken for PMTCT or ART [56].

Cotrimoxazole prophylaxis was administered as a trial intervention to all study participants following recommendations for HIV-infected pregnant women in poor-resource settings [10]. The overall observed adherence to CTXp was high with 75% of the women taking more than 80% of the expected doses. There is very little information on CTXp adherence during pregnancy; however, it is likely to be lower than reported in the context of this clinical trial, and therefore the antimalarial prevention of CTXp in pregnancy and its effectiveness may not be as high in real life situations.

The majority (80%) of HIV-infected women in the world live in Africa, 75% of them being younger than 25 years of age [64]. In some countries hardest hit by the HIV epidemic, as many as 40% of pregnant women are HIV-infected [3],[6]. Many of these women are also exposed to malaria infection and it is estimated that HIV infection adds half a million malaria cases per year in pregnancy [65]. As a result of the general immunosuppression the risks and severity of malaria infection and disease is increased in HIV-infected women, translating into more frequent and severe placental infection as well as the loss of the parity pattern of infection observed in non HIV-infected pregnant women [2],[4]. Protection against malaria in this particularly vulnerable group of the population needs further emphasis. Improved harmonization between the two control programs as well as enhanced integration of both HIV and malaria prevention activities within the reproductive health program would help to address this issue. Moreover, there is an urgent need to find additional drugs for malaria prevention that are not contraindicated with the concomitant administration of the drugs used to prevent opportunistic infections in HIV-infected, malaria-exposed, pregnant women [4].

### Conclusions

These results show that an effective antimalarial drug given as IPTp can offer additional protection against malaria and some associated adverse outcomes when added to CTXp and a LLITN, improving malaria prevention and overall maternal health through reduction in hospital admissions. However, because of its poor tolerability at the dose of 15 mg/kg, MQ is not recommended for IPTp [66]. In addition, the observed association with an increased viral load at delivery and frequency of MTCT of HIV raises further concern about its use in this context. The benefits of improved malaria prevention in this particularly vulnerable group of pregnant women highlight the need for alternative drugs with better tolerability. Moreover, specifically designed studies to fully understand the implications of co-administration of antimalarials and ARVs are needed. Finally, these results may also have implications regarding the antimalarial drug combinations containing MQ currently recommended for malaria treatment.

## Supporting Information

Figure S1 **Trial profile (ATP cohort).**
(TIF)

Figure S2 **Time to first episode of clinical malaria.**
(TIF)

Figure S3 **Adherence to cotrimoxazole prophylaxis by country and treatment group.**
(TIF)

Figure S4 **HIV viral load by country.**
(TIF)

Table S1 **List of regulatory authorities and national ethical review boards.**
(DOC)

Table S2 **Characteristics of study sites.**
(DOC)

Table S3 **Adverse events by treatment group and cotrimoxazole adherence.**
(DOC)

Table S4 **Placental histology results by treatment group.**
(DOCX)

Text S1 **MiPPAD study protocol.**
(PDF)

Text S2 **CONSORT checklist.**
(DOC)

Text S3 **Statistical analytical plan.**
(PDF)

Text S4 **ClinicalTrials.gov registration receipt.**
(PDF)

Text S5 **Ethics approval documentation.**
(PDF)

## Abbreviations

AE: adverse event
ANC: antenatal care
ART: antiretroviral therapy
ARV: antiretroviral
ATP: according to protocol
CTXp: cotrimoxazole prophylaxis
Hb: haemoglobin
IPTp: intermittent preventive treatment in pregnancy
IQR: interquartile range
ITT: intention to treat
LLITN: long lasting insecticide treated net
MQ: mefloquine
MTCT: mother to child transmission
PMTCT: prevention of mother to child transmission
RR: risk ratio
SAE: serious adverse event
SD: standard deviation
SP: sulfadoxine-pyrimethamine
