# Supplementary figures and images for: Intermittent Preventive Treatment of Malaria in Pregnancy with Mefloquine in HIV-Infected Women Receiving Cotrimoxazole Prophylaxis: A Multicenter Randomized Placebo-Controlled Trial
Source: PLoS Med. 2014 Sep 23;11(9):e1001735. doi: 10.1371/journal.pmed.1001735 (PMC4172537; doi:10.1371/journal.pmed.1001735)

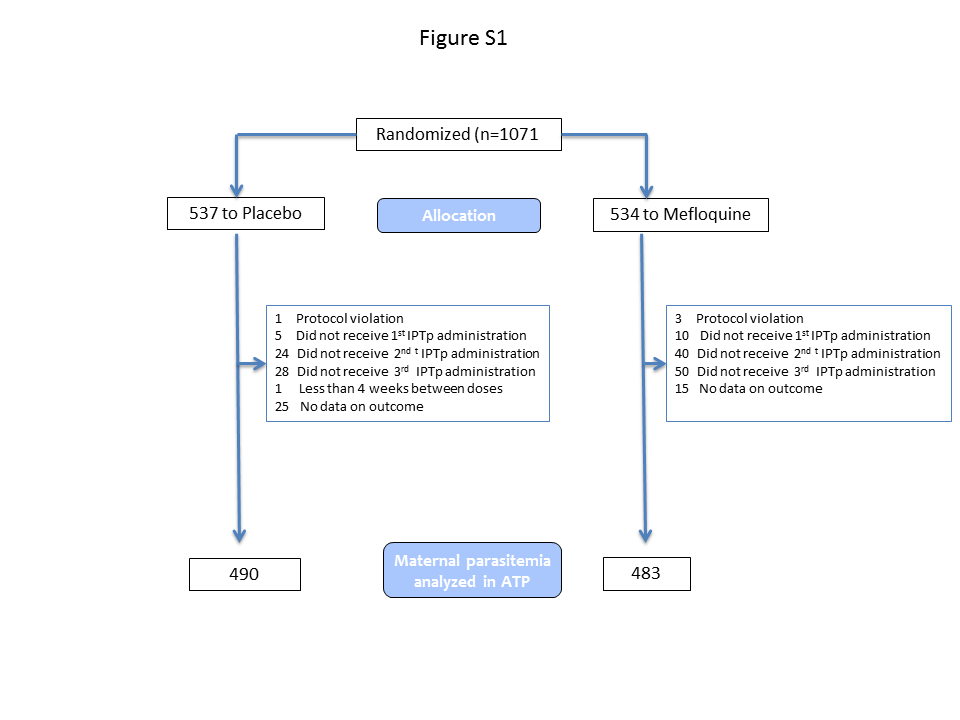

Supplement: Figure S1 — Trial profile (ATP cohort). (TIF) [file pmed.1001735.s001.tif]

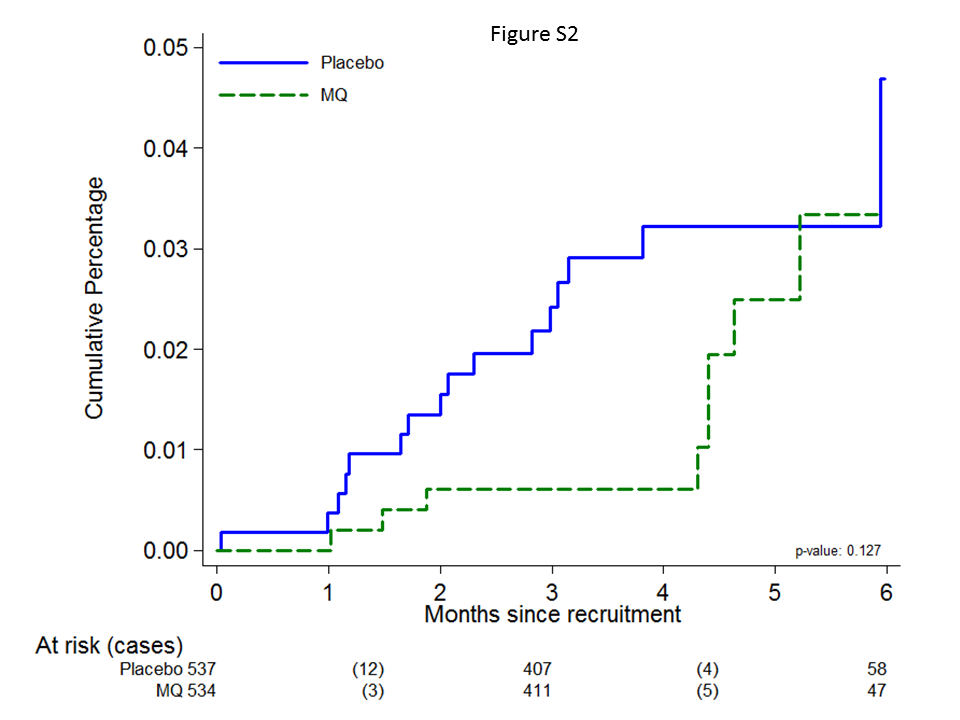

Supplement: Figure S2 — Time to first episode of clinical malaria. (TIF) [file pmed.1001735.s002.tif]

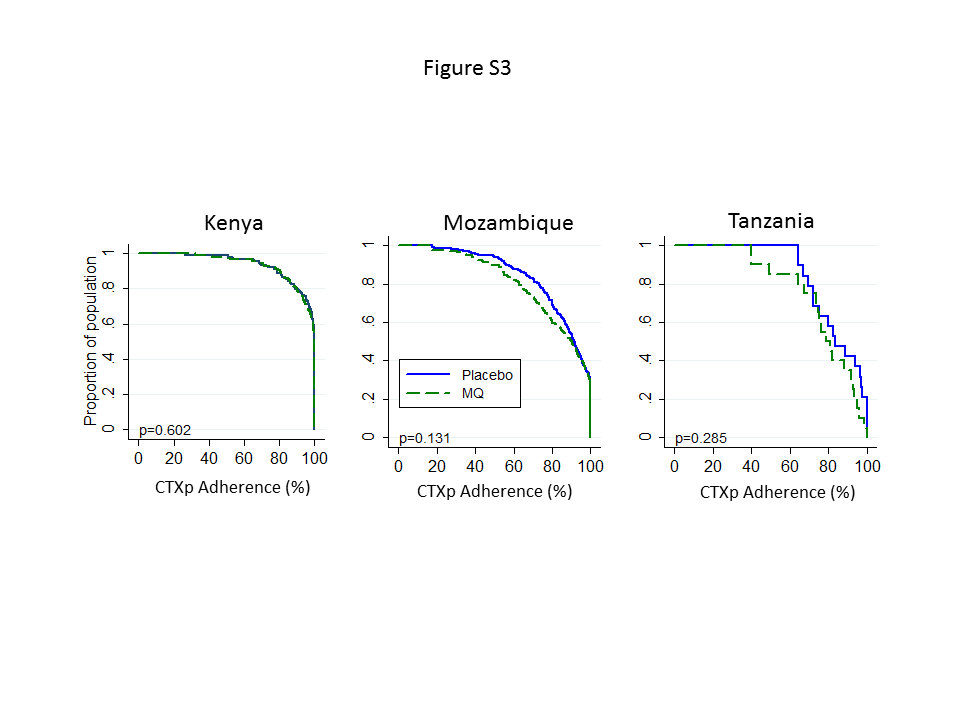

Supplement: Figure S3 — Adherence to cotrimoxazole prophylaxis by country and treatment group. (TIF) [file pmed.1001735.s003.tif]

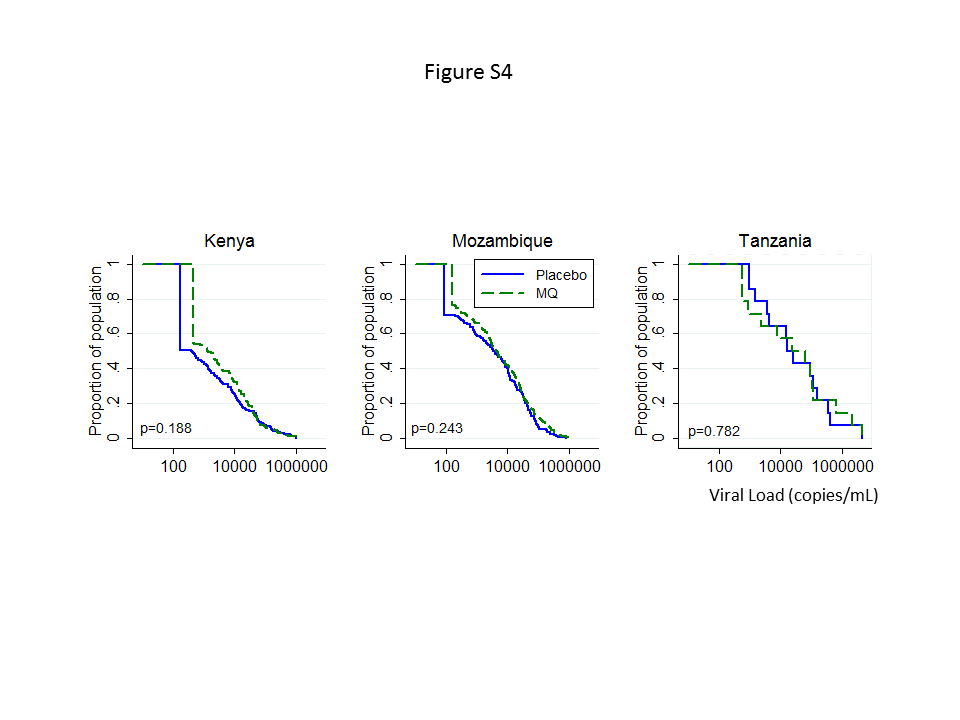

Supplement: Figure S4 — HIV viral load by country. (TIF) [file pmed.1001735.s004.tif]
