# Supplementary material for: Intermittent Preventive Treatment of Malaria in Pregnancy with Mefloquine in HIV-Infected Women Receiving Cotrimoxazole Prophylaxis: A Multicenter Randomized Placebo-Controlled Trial
Source: PLoS Med. 2014 Sep 23;11(9):e1001735. doi: 10.1371/journal.pmed.1001735 (PMC4172537; doi:10.1371/journal.pmed.1001735)
Supplement: Table S1 — List of regulatory authorities and national ethical review boards. (DOC) [file pmed.1001735.s005.doc]

**Table S1. L**ocal regulatory authorities and national ethical review boards

| **Country** | **Review Board** |
| --- | --- |
| **Kenya** | - KEMRI Scientific Steering Committee - KEMRI National Ethics Review Committee - Kenya Pharmacy and Poisons Board (KPPB) |
| **Mozambique** | - Comité Nacional de Bioética para a Saúde (Ministério da Saúde) - Departamento Farmacéutico (Ministério da Saúde ) |
| **Tanzania** | - Institutional Review Board - National Institute for Medical Research Review Board - Tanzania Food and Drug Association |
