# Supplementary material for: Intermittent Preventive Treatment of Malaria in Pregnancy with Mefloquine in HIV-Infected Women Receiving Cotrimoxazole Prophylaxis: A Multicenter Randomized Placebo-Controlled Trial
Source: PLoS Med. 2014 Sep 23;11(9):e1001735. doi: 10.1371/journal.pmed.1001735 (PMC4172537; doi:10.1371/journal.pmed.1001735)
Supplement: Table S2 — Characteristics of study sites. (DOC) [file pmed.1001735.s006.doc]

**Table S2. Characteristics of MiPPAD study sites**

| **Country** | **Kenya** | **Tanzania** | **Mozambique** |
| --- | --- | --- | --- |
| **Sites** | - Siaya | - Makole - Chamwino | - Manhiça - Maragra |
| **Malaria Transmission** | Holoendemic | Mesoendemic | Mesoendemic |
| ***P. falciparum* infection** | >90% | >90% | >90% |
| **High season** | May-Jul | Jun-Aug | Sep-Mar |
| **HIV prevalence in pregnant women** | 18.4%^1^ | 6.9%^3^ | 29%^5^ |
| **Perinatal MTCT of HIV** | 6%^2^ | 15%^4^ | 9%^6^ |
| **HIV tests** | - SD Bioline HIV (Standard Diagnostics, Korea) - Unigold HIV (Trinity Biotech, Ireland) | - Determine (Abbott Lab, USA) - SD Bioline HIV (Standard Diagnostics, Korea) | - Determine (Abbott Lab, USA) - Unigold (TM HIV, Trinity Biotech, Ireland) |
| **Syphilis tests** | - Determine Syphillis TP (Alere Medical Company Limited, Japan) - Determine HIV 1/2  (Alere Medical Company Limited, Japan) | - SD Bio line (Abon Biopharm, Hangzhuo, Co. Ltd) | - SD Bioline Syphilis 3.0 (Standard Diagnostics, Korea) |

^1^Ministry of Health- Republic of Kenya (2012)."Kenya, County HIV Service Delivery Profiles." National AIDS and STI Control Program; ^2^ Ministry of Health- Republic of Kenya (2012). "Early Infant Diagnosis Program. National AIDS and STI Control Program." www.nascop.org/eid [accessed February 2014]; ^3^Ministry of Health and Social Welfare-The United Republic of Tanzania (2008). "Surveillance of HIV and Syphilis Infections Among Antenatal Clinic Attendees." http://pmtct.or.tz/pmtct-tanzania/pmtct-in-tanzania/ [accessed February 2014]; ^4^Ministry of Health and Social Welfare-The United Republic of Tanzania (2013). "UNAIDS 2013 Global Report." http://pmtct.or.tz/pmtct-tanzania/pmtct-in-tanzania/ [accessed February 2014]; ^5^Gonzalez R. et al.(2012) HIV Med; ^6^ Moraleda C. et al. (2014) J Acquir Immune Defic Syndr.
