# Supplementary material for: Intermittent Preventive Treatment of Malaria in Pregnancy with Mefloquine in HIV-Infected Women Receiving Cotrimoxazole Prophylaxis: A Multicenter Randomized Placebo-Controlled Trial
Source: PLoS Med. 2014 Sep 23;11(9):e1001735. doi: 10.1371/journal.pmed.1001735 (PMC4172537; doi:10.1371/journal.pmed.1001735)
Supplement: Table S3 — Adverse events by treatment group and cotrimoxazole adherence. (DOC) [file pmed.1001735.s007.doc]

**Table S3. Adverse events by treatment group and cotrimoxazole** adherence

| **CTXp adherence** | **Control**  **n % 95%CI** | | | **Mefloquine**  **n % 95%CI** | | |
| --- | --- | --- | --- | --- | --- | --- |
| **First tertile of adherence (<87%)** |  | | |  | | |
| 1^st^ IPTp administration |  | | |  | | |
| Dizziness | 13 | 8.18 | 4.43; 13.58 | 52 | 30.23 | 23.47; 37.69 |
| Vomiting | 3 | 1.89 | 0.39; 5.41 | 42 | 24.42 | 18.20; 31.54 |
| 2nd IPTp administration |  | | |  | | |
| Dizziness | 3 | 2.00 | 0.41; 5.73 | 24 | 15.09 | 9.92; 21.62 |
| Vomiting | 4 | 2.67 | 0.73; 6.69 | 23 | 14.47 | 9.40; 20.91 |
| 3rd IPTp administration |  | | |  | | |
| Dizziness | 1 | 0.74 | 0.02; 4.06 | 14 | 10.77 | 6.01; 17.41 |
| Vomiting | 4 | 2.96 | 0.81; 7.41 | 8 | 6.15 | 2.69; 11.77 |
| **Second tertile of adherence (87-<100%)** |  |  |  |  |  |  |
| 1^st^ IPTp administration |  |  |  |  |  |  |
| Dizziness | 9 | 6.92 | 3.21; 12.74 | 31 | 25.20 | 17.81; 33.83 |
| Vomiting | 5 | 3.85 | 1.26;8.75 | 30 | 24.39 | 17.10; 32.95 |
| 2nd IPTp administration |  |  |  |  |  |  |
| Dizziness | 7 | 5.38 | 2.19; 10.78 | 22 | 18.80 | 12.18; 27.07 |
| Vomiting | 1 | 0.77 | 0.02; 4.21 | 18 | 15.38 | 9.38; 23.22 |
| 3rd IPTp administration |  |  |  |  |  |  |
| Dizziness | 3 | 2.40 | 0.50; 6.85 | 10 | 8.85 | 4.33; 15.67 |
| Vomiting | 3 | 2.40 | 0.50; 6.85 | 8 | 7.08 | 3.11; 13.47 |
| **Third tertile of adherence (100%)** |  |  |  |  |  |  |
| 1^st^ IPTp administration |  |  |  |  |  |  |
| Dizziness | 17 | 7.73 | 4.57;12.08 | 67 | 34.01 | 27.43; 41.08 |
| Vomiting | 7 | 3.18 | 1.29;6.45 | 49 | 24.87 | 19.00; 31.52 |
| 2nd IPTp administration |  |  |  |  |  |  |
| Dizziness | 6 | 2.80 | 1.04; 6.00 | 38 | 19.59 | 14.25; 25.88 |
| Vomiting | 5 | 2.34 | 0.76; 5.37 | 32 | 16.49 | 11.56; 22.48 |
| 3rd IPTp administration |  |  |  |  |  |  |
| Dizziness | 5 | 2.49 | 0.81; 5.71 | 17 | 9.39 | 5.57; 14.61 |
| Vomiting | 5 | 2.49 | 0.81; 5.71 | 19 | 10.50 | 6.44; 15.91 |
