# Supplementary material for: Intermittent Preventive Treatment of Malaria in Pregnancy with Mefloquine in HIV-Infected Women Receiving Cotrimoxazole Prophylaxis: A Multicenter Randomized Placebo-Controlled Trial
Source: PLoS Med. 2014 Sep 23;11(9):e1001735. doi: 10.1371/journal.pmed.1001735 (PMC4172537; doi:10.1371/journal.pmed.1001735)
Supplement: Table S4 — Placental histology results by treatment group. (DOCX) [file pmed.1001735.s008.docx]

**Table S4. Placental histology results by treatment**

| Placental Histology | **Control**  **n %** | | **MQ**  **n %** | | **p-value*** |
| --- | --- | --- | --- | --- | --- |
| ITT  Acute infection | 8 | 1.75 | 3 | 0.68 | 0.472 |
| Chronic infection | 5 | 1.09 | 4 | 0.91 |  |
| Past infection | 48 | 10.48 | 42 | 9.50 |  |
| Not infected | 397 | 86.68 | 393 | 88.91 |  |
| ATP  Acute infection | 8 | 1.92 | 3 | 0.78 |  |
| Chronic infection | 4 | 0.96 | 4 | 1.04 | 0.582 |
| Past infection | 44 | 10.58 | 41 | 10.62 |  |
| Not infected | 360 | 86.54 | 338 | 87.56 |  |

* Pearson Chi square
