# Supplementary material for: Intermittent Preventive Treatment of Malaria in Pregnancy with Mefloquine in HIV-Infected Women Receiving Cotrimoxazole Prophylaxis: A Multicenter Randomized Placebo-Controlled Trial
Source: PLoS Med. 2014 Sep 23;11(9):e1001735. doi: 10.1371/journal.pmed.1001735 (PMC4172537; doi:10.1371/journal.pmed.1001735)
Supplement: Text S3 — Statistical analytical plan. (PDF) [file pmed.1001735.s011.pdf]

Analysis Plan

For the MiPPAD trial 2

Author:	MiPPAD Stats-WG
Version:	Final Version 1.0
Date:	10/Jun/2013
Title:	Evaluation of the safety and efficacy of Mefloquine as Intermittent Preventive Treatment for malaria in Pregnancy
Acronym:	MiPPAD (MOT2)

Contents

1 Introduction **6**

2 Objectives **6**
2.1 Primary . . . 6
2.2 Secondary . . . 6

3 Endpoints **6**
3.1 Primary . . . 6
3.2 Secondary . . . 6
3.3 Exploratory . . . 7

4 Study Design Overview **7**

5 Population for analysis **10**
5.1 According to protocol (ATP) . . . 10
5.2 Intention to treat (ITT) . . . 10
5.3 Safety . . . 10

6 Statistical methods **10**

7 Skeleton of tables and graphs **12**
7.1 Trial profile (ATP,ITT,Safety) . . . 12
7.2 Baseline characteristics(ATP,ITT,Safety) . . . 14
7.3 Efficacy (ATP adjusted, ATP crude, ITT) . . . 15
7.3.1 Primary endpoint . . . 15
7.3.2 Secondary endpoints . . . 15
7.4 Safety (ITT,Safety) . . . 17

8 Individual data listings **19**

List of Abbreviations

MiPPAD Malaria in Pregnancy Preventive Alternative Drugs

AP Analysis Plan

CTX Cotrimoxazol

IQR Interquartilic range

LLITNs Long lasting insecticide treated nets

MQ Mefloquine

MUAC Mid-upper arm circumference

RR Risk Ratio

RRatio Rate Ratio

List of Tables

1 Maternal visits and procedures schedule . . . 8
2 Children visits . . . 9
3 Trial profile . . . 12
4 Reasons for Withdrawn before dose 1 . . . 12
5 Reasons for Withdrawn before dose 2 . . . 12
6 Reasons for Withdrawn before dose 3 . . . 13
7 Reasons for Withdrawn before delivery . . . 13
8 Reasons for Withdrawn before completing follow-up . . . 13
9 Baseline characteristics . . . 14
10 Peripheral parasitaemia at delivery by treatment group . . . 15
11 Secondary endpoints (endpoints for prevalence) . . . 15
12 Secondary endpoints (endpoints for continous variables) . . . 16
13 Secondary endpoints (endpoints for incidences) . . . 16
14 Secondary endpoints (endpoints for neonatal mortality) . . . 16
15 Incidence of serious adverse events classified by MedDRA . . . 17
16 Incidence of adverse pregnancy outcomes . . . 17
17 Incidence of adverse events . . . 17
18 Incidence of adverse events related to medication . . . 18

List of Figures

1 Introduction

This Analysis Plan (AP) provides detailed description of the descriptive (with the skeleton of the tables) and inferential statistical analysis for the efficacy and safety data collected in the Trial 2 of the MiPPAD study to compare the IPT_p-MQ versus IPT_p-Placebo in HIV infected women receiving Cotrimoxazol (CTX) and Long lasting insectide treated nets (LLITNs). A Separate AP is available for the analysis of exploratory endpoints and the analysis of children,

Any planned modification of the analysis should be documented in this AP.

2 Objectives

2.1 Primary

To determine the safety and efficacy of IPTp with mefloquine among HIV infected women receiving CTX prophylaxis for opportunistic infections

2.2 Secondary

- To evaluate the efficacy of CTX in the prevention of malaria infection in pregnant women.
- To compare immune status of HIV infected women receiving CTX + IPTp-MQ to those receiving CTX + IPTp-placebo.
- To assess the safety of study drugs in the development of infants

3 Endpoints

3.1 Primary

- Prevalence of peripheral parasitaemia (microscopic and submicroscopic) at delivery

3.2 Secondary

- Prevalence of *P. falciparum* parasitaemia in cord blood (microscopic and submicroscopic[1])
- Prevalence of placental *P. falciparum* infection (histology or blood smear, submicroscopic [1])
- Mean maternal haemoglobin (g/dL) at delivery
- Prevalence of maternal anaemia at delivery (<11 g/dL)
- Prevalence of severe maternal anaemia at delivery (<7 g/dL)
- Mean of CD4 counts and viral load at delivery
- Prevalence of neonatal anemia (Hb<12.5 g/dL in cas e of cord blood, or Hb<13g/dL in case of peripheral blood)
- Mean birth weight (in grams)

[1]Only in a subsample of participants

- Prevalence of low birth weight babies (<2500 g)
- Prevalence of prematurity
- Number of stillbirths
- Number of miscarriages
- Frequency of congenital malformations
- Mean gestational age at birth (assessed by Ballard Score in the newborn)
- Incidence of vomiting
- Incidence of dizziness
- Frequency of drug adverse reactions
- Incidence of clinical malaria during pregnancy
- Incidence of overall admissions/outpatient attendances
- Neonatal mortality
- Perinatal mortality
- Peripheral maternal parasitemia 1 month[2] after end of pregnancy (microscopic and submicroscopic[1])

3.3 Exploratory

- Congenital malaria
- Pharmacovigilance
- SAEs in chidren

4 Study Design Overview

This is a randomized double-blind superiority clinical trial to compare the efficacy of MQ as IPTp (IPT_p-MQ) with placebo-IPTp (IPT_p-Placebo) in HIV-infected pregnant women receiving CTX prophylaxis.The table 1 shows the visits and procedures schedule to the mother and the table 2 shows the visits and procedures schedule to the child.

[2] At visit5, aproximately 1 month after end of pregnancy

Table 1: Maternal visits and procedures schedule

Study Procedures	Visit 1 Enrollment	Visit 2	Visit 3	Visit 4 Delivery	Visit 5 1 Month	Unscheduled visits	Household visits
Inclusion/Exclusion criteria	X						
Written infomed consent	X						
Demographics/Medical history	X						
Record of concomitant medication/ Adverse events	X	X	X	X	X	X	
Physical examination / Clinical	X					X	
Gestational Age	X	X	X	X		X	
Temperature					X	X	
Blood Pressure				X		X	
Weight	X	X	X			X	
Height	X						
MUAC	X						
IPTp Administration	X	X	X				
CTX Administration	X	*	*	*		*	*
RPR test	X						
HIV test	X						
CD4 Count	X						
Viral load	X						
Blood smear	†	†	†	X	X	†	
Haemoglobin test	X			X	X		
Peripheral venous blood (mother)				X			
Cord blod sample				X			
Placental biopsy				X			
Placental impression smears				X			
Drug tolerability assesment	X	X	X				X
LLITNs	X	‡	‡	‡	‡	‡	‡

*CTX Adherence should be assessed at each scheduled visit.
†Only in women passively reporting sick AND presenting with malaria related signs/symptoms (fever $\geq 37.5^{\circ}$C).
‡Assesment of compliance with LLITNs.

Table 2: Children visits

Study Procedure	At Birth	1 month*	2 months	Unscheduled visits
Medical history	X	X	X	X
Physical examination	X	X	X	X
PsycHomotor development assesment	X	X	X	
Weight	X	X	X	X
Height	X	X	X	X
Temperature	X	X	X	X
Blood smear	X	†	†	†
Haemoglobin test	X	†	†	†
HIV PCR §		X		

* First visit will be scheduled 1 monthafter birth or coinciding with 1st EPI visit.

†Only if fever ($\geq$ 37.5°C) or history of fever in the past 24 hours or signs suggestive of malaria.

§HIV PCR test should be repeated at month 18 after birth.

5 Population for analysis

The assignment of participants into analysis populations will be performed prior to database lock and any data analysis. The following populations are defined:

5.1 According to protocol (ATP)

This population includes all women who fulfill all the inclusion-exclusion criteria and took the three IPT doses, received the LLITN, received prophylaxis with CTX and from whom data is available for the analysis. Subjects will be excluded from the ATP population in case of:

- No fulfill inclusion criteria
- No data on outcome
- No three doses of IPTp
- Wrong study drug administered
- Do not receive LLITN
- Less than 4 weeks (28 days) between IPTp doses

5.2 Intention to treat (ITT)

This population includes all randomized women. Following the intention-to-treat principle, patients will be analyzed according to the preventive treatment they were assigned to at randomization. This population is the target population for the efficacy analysis

5.3 Safety

This population includes all patients who received at least one dose of IPT and had at least one post-baseline safety assessment. Patients will be analyzed according to preventive treatment assigned. This is the target population for the safety and tolerability analysis

6 Statistical methods

The primary analysis of this trial is the comparison of the proportion of mothers with peripheral parasitaemia at delivery in the ATP cohort, adjusted by gravity, country, seasonality and other variables associated with the prevalence of peripheral parasitemia at delivery.

Proportions are compared between group using fisher exact test and presented as relative risk ratio (RR) or reduction of the RR ($1 - RR * 100\%$) if RR lower than 1. Adjustment for co-variates and possible confounder are done using poisson regression with a log link and robust estimate of the covariance (Huber method), using the method proposed by Zou (A modified poisson regression approach to prospective studies with binary data, American Journal of Epidemiology, 2004:159(7): 702-706)

Continuous variables are compared between groups using Wilcoxon rank sum test and the effect presented as Mean Difference. Adjustment for co variates and possible con founders are done using ordinary least square regression. Variables will be transformed to the logarithm scale if normality is improved and result presented as Proportional Difference. If after transformation, non-normality of the residuals is

detected using diagnostic regression plots (q-q plots of the residuals, and plot of the residuals against the predicted values), robust intervals of confidence and Wald test will be resented instead.

Incidence of clinical malaria, overall admissions and outpatient attendances will be estimated as the number of episodes over the time at risk. Time at risk is estimated as the time from the start of follow up (dose 1 in mothers) until the end of follow-up (visit one month after deliver for mothers) or withdrawal due to censoring or death, whatever occurs first. In order to avoid to count twice the same clinical malaria episode, subjects will not contribute to the denominator nor the numerator during an arbitrary period of 28 days after episode of clinical malaria is defined. A maximum of one episode of admission or outpatient visit will be count per day. The total number of events will be compare between groups using Negative Binomial regression models which take into account a possible extra Poisson variation due to different frailty of the subjects. The comparison will be expressed as relative rate ratio (RRate).

To calculate the compliance with CTX medication, the number of tables taken (from number of tables given minus the number of tablets returned) will be related to the number of days under chemoprophylaxis (from date of deliver - date of first dose of CTX given). A Mother will be considered compliant if more than 80% of the expected medication is taken.

7 Skeleton of tables and graphs

7.1 Trial profile (ATP,ITT,Safety)

Table 3: Trial profile

Screened	#	
Group	IPT_p-MQ	IPT_p-Placebo
Randomized	#	#
Receives ITN	#	#
Withdrawn before dose 1	#	#
Dose 1 IPTp	#	#
Withdrawn before dose 2	#	#
Dose 2 IPTp	#	#
Withdrawn before dose 3	#	#
Dose 3 IPTp	#	#
Withdrawn before deliver	#	#
Delivery	#	#
Withdrawn before completing follow-up	#	#
Visit after delivery	#	#

Table 4: Reasons for Withdrawn before dose 1

	IPT_p-MQ	IPT_p-Placebo
Serious Adverse Events	#	#
Non Serious Adverse Events	#	#
Protocol Violation	#	#
Consent withdrawal	#	#
Migrated/moved from study area	#	#
Lost to follow-up	#	#
Others	#	#

Table 5: Reasons for Withdrawn before dose 2

	IPT_p-MQ	IPT_p-Placebo
Serious Adverse Events	#	#
Non Serious Adverse Events	#	#
Protocol Violation	#	#
Consent withdrawal	#	#
Migrated/moved from study area	#	#
Lost to follow-up	#	#
Others	#	#

Table 6: Reasons for Withdrawn before dose 3

	IPT_p-MQ	IPT_p-Placebo
Serious Adverse Events	#	#
Non Serious Adverse Events	#	#
Protocol Violation	#	#
Consent withdrawal	#	#
Migrated/moved from study area	#	#
Lost to follow-up	#	#
Others	#	#

Table 7: Reasons for Withdrawn before delivery

	IPT_p-MQ	IPT_p-Placebo
Serious Adverse Events	#	#
Non Serious Adverse Events	#	#
Protocol Violation	#	#
Consent withdrawal	#	#
Migrated/moved from study area	#	#
Lost to follow-up	#	#
Others	#	#

Table 8: Reasons for Withdrawn before completing follow-up

	IPT_p-MQ	IPT_p-Placebo
Serious Adverse Events	#	#
Non Serious Adverse Events	#	#
Protocol Violation	#	#
Consent withdrawal	#	#
Migrated/moved from study area	#	#
Lost to follow-up	#	#
Others	#	#

7.2 Baseline characteristics(ATP,ITT,Safety)

Table 9: Baseline characteristics

Variable		IPT_p-MQ (N=XXX)	IPT_p-Placebo (N=XXX)
Country (n(%))	Kenya	#(#.#%)	#(#.#%)
	Mozambique	#(#.#%)	#(#.#%)
	Tanzania	#(#.#%)	#(#.#%)
Age (years, mean(sd))		#(#.#)	#(#.#)
Parity (n(%))	Primigravidae	#(#.#%)	#(#.#%)
	1 to 3 previous pregnancies	#(#.#%)	#(#.#%)
	4 or more	#(#.#%)	#(#.#%)
Weight (kg, mean(sd))		#(#.#)	#(#.#)
Height (cm, mean(sd))		#(#.#)	#(#.#)
MUAC (cm, mean(sd))		#(#.#)	#(#.#)
Gestational age (weeks, median(IQR))		#(#:#)	#(#:#)
Gestational age in categories	First trimester(0-12 weeks)	#(#.#%)	#(#.#%)
	Second trimester(12-24 weeks)	#(#.#%)	#(#.#%)
	Third trimester($\geq$ 25 weeks)	#(#.#%)	#(#.#%)
Literate (can read and/or write, n(%))		#(#.#%)	#(#.#%)
RPR test (Positive, n(%))		#(#.#%)	#(#.#%)
Hemoglobin (g/dl, mean(sd))		#(#.#)	#(#.#)
Anemia (Hb <; 11, n(%))		#(#.#%)	#(#.#%)
Viral Load	Indetectable or < 400	#(#.#%)	#(#.#%)
	low (400-10^3)	#(#.#%)	#(#.#%)
	Medimum ($10^3 < 10^5$)	#(#.#%)	#(#.#%)
	High ($> 10^5$)	#(#.#%)	#(#.#%)
CD4 T-cell counts $\leq$ 350 cells/μl		#(#.#%)	#(#.#%)
On HAART at recruitment		#(#.#%)	#(#.#%)
Start HAART after recruitment		#(#.#%)	#(#.#%)
Receive AZT during pregnancy		#(#.#%)	#(#.#%)
Receive 3TC during pregnancy		#(#.#%)	#(#.#%)
Receive NVP during pregnancy		#(#.#%)	#(#.#%)
Receive AZT/NVP at delivery		#(#.#%)	#(#.#%)
New born receive NVP at delivery		#(#.#%)	#(#.#%)
Post-deliver PMTCT Prophylaxis to the mother		#(#.#%)	#(#.#%)
Post-deliver PMTCT Prophylaxis to the child		#(#.#%)	#(#.#%)

N: Number of subjects in the group, sd: Standard deviation,
n: Number of subjects in the category IQR: Interquartilic range,
%: Percentage

7.3 Efficacy (ATP adjusted, ATP crude, ITT)

7.3.1 Primary endpoint

Table 10: Peripheral parasitaemia at delivery by treatment group

Variable	Group	N	n(%)	RR (95% CI)	p-value
Peripheral parasitaemia at delivery	IPT_p-MQ<br>IPT_p-Placebo	###<br>###	## (##.#)<br>## (##.#)	#.## (#.##; #.##)	#.###

RR:Risk Ratio, N: Number of subject per group, n: number of subjects in the category.

7.3.2 Secondary endpoints

Table 11: Secondary endpoints (endpoints for prevalence)

Variable	Group	N	n(%)	RR (95% CI)	p-value
Placental *P. falciparum* infection	IPT_p-MQ<br>IPT_p-Placebo	###<br>###	## (##.#)<br>## (##.#)	#.## (#.##; #.##)	#.###
Low birth weight babies (< 2500)	IPT_p-MQ<br>IPT_p-Placebo	###<br>###	## (##.#)<br>## (##.#)	#.## (#.##; #.##)	#.###
Pre term babies	IPT_p-MQ<br>IPT_p-Placebo	###<br>###	## (##.#)<br>## (##.#)	#.## (#.##; #.##)	#.###
Fetal anaemia	IPT_p-MQ<br>IPT_p-Placebo	###<br>###	## (##.#)<br>## (##.#)	#.## (#.##; #.##)	#.###
Maternal anaemia at delivery	IPT_p-MQ<br>IPT_p-Placebo	###<br>###	## (##.#)<br>## (##.#)	#.## (#.##; #.##)	#.###
Severe maternal anemia at delivery	IPT_p-MQ<br>IPT_p-Placebo	###<br>###	## (##.#)<br>## (##.#)	#.## (#.##; #.##)	#.###
Peripheral maternal microscopic parasitaemia at delivery	IPT_p-MQ<br>IPT_p-Placebo	###<br>###	## (##.#)<br>## (##.#)	#.## (#.##; #.##)	#.###
Peripheral maternal submicroscopic parasitaemia at delivery	IPT_p-MQ<br>IPT_p-Placebo	###<br>###	## (##.#)<br>## (##.#)	#.## (#.##; #.##)	#.###
Cord blood microscopic parasitaemia at delivery	IPT_p-MQ<br>IPT_p-Placebo	###<br>###	## (##.#)<br>## (##.#)	#.## (#.##; #.##)	#.###
Cord blood submicroscopic parasitaemia at delivery	IPT_p-MQ<br>IPT_p-Placebo	###<br>###	## (##.#)<br>## (##.#)	#.## (#.##; #.##)	#.###
Stillbirths	IPT_p-MQ<br>IPT_p-Placebo	###<br>###	## (##.#)<br>## (##.#)	#.## (#.##; #.##)	#.###

RR: Risk ratio, N: Number of subject per group, n: number of subjects in the category.

Table 12: Secondary endpoints (endpoints for continous variables)

Variable	Group	N	mean	sd	Diff (95% CI)	p-value
CD4 Counts (per μl) at delivery	IPT_p-MQ<br>IPT_p-Placebo	###<br>###	##.#<br>##.#	##.#<br>##.#	#.## (#.##; #.##)	#.###
Viral load (. . .)	IPT_p-MQ<br>IPT_p-Placebo	###<br>###	##.#<br>##.#	##.#<br>##.#	#.## (#.##; #.##)	#.###
Maternal haemoglobin (g(dL))	IPT_p-MQ<br>IPT_p-Placebo	###<br>###	##.#<br>##.#	##.#<br>##.#	#.## (#.##; #.##)	#.###
Foetal haemoglobin (g(dL))	IPT_p-MQ<br>IPT_p-Placebo	###<br>###	##.#<br>##.#	##.#<br>##.#	#.## (#.##; #.##)	#.###
Birth weight (gr))	IPT_p-MQ<br>IPT_p-Placebo	###<br>###	##.#<br>##.#	##.#<br>##.#	#.## (#.##; #.##)	#.###
Variable	Group	N	Geometric mean	IRQ	PDiff (95% CI)	p-value
Gestational age (weeks) *when..*	IPT_p-MQ<br>IPT_p-Placebo	###<br>###	##.#<br>##.#	##.#:##.#<br>##.#: ##.#	#.## (#.##; #.##)	#.###

Diff = difference betweeng groups. PDiff= Proportional difference between group. IRQ: Interquartilic range

Table 13: Secondary endpoints (endpoints for incidences)

Variable	Group	N	Events	PYAR	Incidence	RR (95% CI)	p-value
Clinical malaria during pregnancy	IPT_p-MQ<br>IPT_p-Placebo	###<br>###	##<br>##	###<br>###	#.###<br>#.###	#.## (#.##; #.##)	#.###
Overall maternal admisions	IPT_p-MQ<br>IPT_p-Placebo	###<br>###	##<br>##	###<br>###	#.###<br>#.###	#.## (#.##; #.##)	#.###
Maternal outpatient visits	IPT_p-MQ<br>IPT_p-Placebo	###<br>###	##<br>##	###<br>###	#.###<br>#.###	#.## (#.##; #.##)	#.###

RRatio: Relative Ratio, N: Number of subject per group, PYAR: Person years at risk.

Table 14: Secondary endpoints (endpoints for neonatal mortality)

Variable	Group	N	n(%)	RR (95% CI)	p-value
Perinatal Mortality	IPT_p-MQ<br>IPT_p-Placebo	###<br>###	## (##.#)<br>## (##.#)	#.## (#.##; #.##)	#.###
Neonatal moratality	IPT_p-MQ<br>IPT_p-Placebo	###<br>###	## (##.#)<br>## (##.#)	#.## (#.##; #.##)	#.###
Early neonatal mortality	IPT_p-MQ<br>IPT_p-Placebo	###<br>###	## (##.#)<br>## (##.#)	#.## (#.##; #.##)	#.###

RR: Risk ratio, N: Number of subject per group, n: number of subjects in the category.

7.4 Safety (ITT,Safety)

Table 15: Incidence of serious adverse events classified by MedDRA

System Organ Class (code)	Preferred Term (code)	IPT_p-MQ (N= XXX) n	%	(95% CI)	IPT_p-Placebo (N= XXX) n	%	(95% CI)
XXXXXXX XXXXXX (#######)	XXXXXX XXXXXXX (#######)	#	##.#%	(###%; ###%)	#	##.#%	(###%; ###%)
XXXXXXXXX XXXX (#######)	XXXXXX XXXXXXX (#######)	#	##.#%	(###%; ###%)	#	##.#%	(###%; ###%)

N= Number of subjects in the group. n= Number of subjects with at least one symptom. CI= Confidence Interval.

Table 16: Incidence of adverse pregnancy outcomes

Variable	IPT_p-MQ (N= XXX) n	%	(95% CI)	IPT_p-Placebo (N= XXX) n	%	(95% CI)
Prematurity	#	##.#%	(###%; ###%)	#	##.#%	(###%; ###%)
Stillbirths	#	##.#%	(###%; ###%)	#	##.#%	(###%; ###%)
Miscarriages	#	##.#%	(###%; ###%)	#	##.#%	(###%; ###%)
Congenital Malformations	#	##.#%	(###%; ###%)	#	##.#%	(###%; ###%)

N= Number of subjects in the group. n= Number of subjects with at least one symptom. CI= Confidence Interval.

Table 17: Incidence of adverse events

Variable	IPT_p-MQ (N= XXX) n	%	(95% CI)	IPT_p-Placebo (N= XXX) n	%	(95% CI)
Any Adverse event	#	##.#%	(###%; ###%)	#	##.#%	(###%; ###%)
Adverse event of grade 3	#	##.#%	(###%; ###%)	#	##.#%	(###%; ###%)
Most frequent AE	#	##.#%	(###%; ###%)	#	##.#%	(###%; ###%)
Second frequent AE	#	##.#%	(###%; ###%)	#	##.#%	(###%; ###%)
Others AE ...	#	##.#%	(###%; ###%)	#	##.#%	(###%; ###%)

N= Number of subjects in the group. n= Number of subjects with at least one symptom. CI= Confidence Interval.

Table 18: Incidence of adverse events related to medication

Variable	IPT_p-MQ (N= XXX)			IPT_p-Placebo (N= XXX)		
	n	%	(95% CI)	n	%	(95% CI)
Any Adverse event	#	##.#%	(###%; ###%)	#	##.#%	(###%; ###%)
Adverse event of grade 3	#	##.#%	(###%; ###%)	#	##.#%	(###%; ###%)
Most frequent AE	#	##.#%	(###%; ###%)	#	##.#%	(###%; ###%)
Second frequent AE	#	##.#%	(###%; ###%)	#	##.#%	(###%; ###%)
Others AE ...	#	##.#%	(###%; ###%)	#	##.#%	(###%; ###%)

N= Number of subjects in the group. n= Number of subjects with at least one symptom. CI= Confidence Interval.

8 Individual data listings

- List of subjects and dates of recruitment, dose1, dose2, delivery,
- List of subjects, treatment and delivery outcome , weight, gestational age
- List of subjects and AEs
- List of subjects and SAE
- ...
