# Supplementary material for: Intermittent Preventive Treatment of Malaria in Pregnancy with Mefloquine in HIV-Infected Women Receiving Cotrimoxazole Prophylaxis: A Multicenter Randomized Placebo-Controlled Trial
Source: PLoS Med. 2014 Sep 23;11(9):e1001735. doi: 10.1371/journal.pmed.1001735 (PMC4172537; doi:10.1371/journal.pmed.1001735)
Supplement: Text S5 — Ethics approval documentation. (PDF) [file pmed.1001735.s013.pdf]

THE UNITED REPUBLIC OF
TANZANIA

National Institute for Medical Research
P.O. Box 9653
Dar es Salaam
Tel: 255 22 2121400/390
Fax: 255 22 2121380/2121360
E-mail: headquarters@nimr.or.tz
NIMR/HQ/R.8c/Vol. 1/119

Ministry of Health and Social Welfare
P.O. Box 9083
Dar es Salaam
Tel: 255 22 2120262-7
Fax: 255 22 2110986

23rd June 2010

Dr Abdunoor Mulokozi
Ifakara Health Institute
P O Box 78373
DAR ES SALAAM

APPROVAL FOR PROTOCOL AMENDMENT

This letter is to confirm that your application for an amendment 02 on the already approved protocol on: Evaluation of the Safety and Efficacy of Mefloquine as intermittent preventive treatment in pregnancy, (Mulokozi A *et al*). Reference NIMR/HQ/R.8a/Vol. IX/804, dated 21st April 2009, NIMR/HQ/R.8c/Vol. 1 /78, 23rd July 2009, has been granted ethics clearance to be conducted in Tanzania.

The Principal Investigator is Dr Abdunoor Mulokozi, IHI, must ensure that the approval is for Amendment 02 Amendment Date is 21 January 2010: To include the changes after DSMB meeting in Barcelona October 2009.

Amendments are:

1. **Protocol version 03 1st October 2009**

2. **A PRIMARY CHANGES:**
   A.1 Statement of the study hypothesis
   A.2 Rearrangement of the Study Objectives
   A.3 Trial design to include the term superiority clinical trial
   A.4 Secondary end points: The number of still births has been added as a secondary endpoint of trial 1 and trial 2.
   A.5 Selection of study participants: The wording of the inclusion procedures of the study participants
   A.6 Administration of IPTp The DSMB suggested to observe all study participants for 60 minutes after administration of the IPTp
   A.7 SAEs reporting
   The timing of SAEs notification and notification has been amended
   A.8 Withdrawal criteria
   In section 5.4 page 32 the word "excluded has been changed to "Withdrawn"
   A.9 Statistical models and methods of analysis

It has been added that MQ efficacy to prevent LBW is deemed to be equal in the 2 MQ groups (full dose and split dose)

3. **OTHER SECONDARY CHANGES**

B. 1 Follow up of study participants

B.2 Informed Consent Trial 1

All changes to the study protocol have been included in the new protocol version 03 -01st October 2009. Other conditions remain as per the original approval.

Approval is valid until 20th April 2011.

Name: Dr Mwelecele Malecela

Signature

ACTING CHAIRPERSON
MEDICAL RESEARCH
COORDNATING COMMITTEE

Name: Dr Deo Mtasiwa

Signature

CHIEF MEDICAL OFFICER
MINISTRY OF HEALTH
AND SOCIAL WELFARE

RMO
DMO

TANZANIA FOOD AND DRUGS AUTHORITY

E-Mail: info@tfda.or.tz
Telephone: +255 22 2450512, 2450751
+255 22 2452108
Fax No. +255 22 2450793
Website: www.tfda.or.tz
All letters should be addressed to
the Director General
In reply please quote Our Ref No:

TFDA
Tanzania Food & Drugs Authority

Nelson Mandela Road,
EPI - Mabibo External,
P.O. Box 77150,
DAR ES SALAAM,
TANZANIA.

Ref. No. CE.57/180/04A/07 **29th June 2010**

Dr. Abdunoor Mulokozi Kabanywanyi
Ifakara Health Institute
P.O.Box 78373
DAR ES SALAAM

RE: APPROVAL TO CONDUCT A STUDY ENTITLED "EVALUATION OF THE SAFETY AND EFFICACY OF MEFLOQUINE AS INTERMITTENT PREVENTIVE TREATMENT FOR MALARIA IN PREGNANCY"

Approval is hereby granted for you to continue conducting above study with **Protocol No.EDCTP-IP.07.31080.002 version 3** dated **01st October 2009.**

The approved study sites are **Ifakara Health Institute (IHI) Dodoma, Dodoma Regional Hospital, Makole Health Centre and Chamwino Dispensary**.

The approval is subject to the following conditions;

1. Complying with the approved protocol.

2. If for any reason the trial is prematurely terminated or suspended, a detailed written explanation must be submitted to TFDA within 21 days.

3. The Authority may withdraw the approval already given if it is dissatisfied with the conduct of study or there are breaches of any conditions prescribed in this letter or law provision.

4. Six monthly progress and final reports should be submitted to TFDA, including interim analyses done by the Data Safety Monitoring Board (DSMB) or related Committee. The progress reports should be submitted within three weeks after the end of the period being reported and the final report within 60 days of conclusion of the trial.

5. All relevant documents and records pertaining to the trial should be retained for a period of at least 3 years after the completion of the trial and made available upon request by TFDA.

6. Any amendment of the protocol, product or investigators brochure should be reported to TFDA and approval obtained before its implementation.

7. All serious adverse events should be reported within two weeks and for fatal ones within 24 hours of their occurrence in any of the study sites.

MISSION
To protect and promote public health by ensuring quality, safety and effectiveness of food, drugs, cosmetics and medical devices

8. Copies of publications of any part of the study should be submitted.

9. The study participants should be insured before the study commences and copies of insurance cover submitted to this office.

Looking forward to your continued cooperation.

Charys Ugullum
Ag. DIRECTOR GENERAL

C.c Clara Menendez
Barcelona Centre for International Health Research,
C/Rossello 132, 4a, 08036 Barcelona,
SPAIN

CU/mf/hi/am

THE UNITED REPUBLIC OF
TANZANIA

National Institute for Medical Research
P.O. Box 9653
Dar es Salaam
Tel: 255 22 2121400/390
Fax: 255 22 2121380/2121360
E-mail: headquarters@nimr.or.tz
NIMR/HQ/R.8a/Vol. IX/804

Ministry of Health and Social Welfare
P.O. Box 9083
Dar es Salaam
Tel: 255 22 2120262-7
Fax: 255 22 2110986

21st April 2009

Dr Abdunoor Mulokozi
Ifakara Health Institute
P O Box 78373
DAR ES SALAAM

CLEARANCE CERTIFICATE FOR CONDUCTING MEDICAL RESEARCH IN TANZANIA

This is to certify that the research entitled: Evaluation of the Safety and Efficacy of Mefloquine as intermittent preventive treatment in pregnancy, (Mulokozi A *et al*), has been granted ethics clearance to be conducted in Tanzania.

The Principal Investigator of the study must ensure that the following conditions are fulfilled:

1. Progress report is made available to the Ministry of Health and the National Institute for Medical Research, Regional and District Medical Officers after every six months.
2. Permission to publish the results is obtained from National Institute for Medical Research.
3. Copies of final publications are made available to the Ministry of Health and the National Institute for Medical Research.
4. Any researcher, who contravenes or fails to comply with these conditions, shall be guilty of an offence and shall be liable on conviction to a fine.
5. Approval is for one year: 21st April 2009 to 20th April 2010.

Name: Dr Andrew Y Kitua

Signature

CHAIRMAN
MEDICAL RESEARCH
COORDINATING COMMITTEE

Name: Dr Deo M Mtasiwa

Signature

CHIEF MEDICA L OFFICER
MINISTRY OF HEALTH, SOCIAL
WELFARE

CC: RMO
DMO

THE UNITED REPUBLIC OF
TANZANIA

National Institute for Medical Research
P.O. Box 9653
Dar es Salaam
Tel: 255 22 2121400/390
Fax: 255 22 2121380/2121360
E-mail: headquarters@nimr.or.tz

Ministry of Health and Social Welfare
P.O. Box 9083
Dar es Salaam
Tel: 255 22 2120262-7
Fax: 255 22 2110986

NIMR/HQ/R.8c/Vol. II/10

24th June 2010

Dr Abdunoor Mulokozi
Ifakara Health Institute
P.O.BOX 78373
DAR ES SALAAM

APPROVAL FOR EXTENSION OF ETHICAL CLEARANCE

This letter is to confirm that your application for extension on the already approved proposal. Evaluation of the Safety and Efficacy of Mefloquine as intermittent preventive treatment in pregnancy, (Mulokozi *A et al*), has been granted ethics clearance to be conducted in Tanzania.

The extension approval is based on the progress report dated 17th May 2010 on the project, Ref NIMR/HQ/R.8A Vol. IX/804. Extension approval is valid until 2o th April 2011.

The Principal Investigator must ensure that other conditions of approval remain as per ethical clearance letter. The PI should ensure that progress and final reports are submitted in a timely manner.

Name: Dr Mwelecele Malecela

Signature

ACTING CHAIRPERSON
MEDICAL RESEARCH
COORDNATING COMMITTEE

Name: Dr Deo Mtasiwa

Signature

CHIEF MEDICAL OFFICER
MINISTRY OF HEALTH
AND SOCIAL WELFARE

RMO
DMO

THE UNITED REPUBLIC OF
TANZANIA

National Institute for Medical Research
P.O. Box 9653
Dar es Salaam
Tel: 255 22 2121400/390
Fax: 255 22 2121380/2121360
E-mail: headquarters@nimr.or.tz

Ministry of Health and Social Welfare
P.O. Box 9083
Dar es Salaam
Tel: 255 22 2120262-7
Fax: 255 22 2110986

NIMR/HQ/R.8c/Vol. II /35

27th April 2011

Dr Abdunoor Mulokozi
Ifakara Health Institute
P.O.BOX 78373
DAR ES SALAAM

APPROVAL FOR EXTENSION OF ETHICAL CLEARANCE

This letter is to confirm that your application for extension on the already approved proposal: Evaluation of the Safety and Efficacy of Mefloquine as intermittent preventive treatment in pregnancy, (Mulokozi A et *al*), has been granted ethics clearance to be conducted in Tanzania.

The extension approval is based on the progress report dated 05th April, 2011 on the project, Ref NIMR/HQ/R.8c/ Vol. II/10. Extension approval is valid until 19th April 2012.

The Principal Investigator must ensure that other conditions of approval remain as per ethical clearance letter. The PI should ensure that progress and final reports are submitted in a timely manner.

Name: Dr Mwelecele Malecela

Signature

ACTING CHAIRPERSON
MEDICAL RESEARCH
COORDNATING COMMITTEE

Name: Dr Deo Mtasiwa

Signature

CHIEF MEDICAL OFFICER
MINISTRY OF HEALTH
AND SOCIAL WELFARE

RMO
DMO

IFAKARA HEALTH INSTITUTE
research | training | services

INSTITUTIONAL REVIEW BOARD
P.O.BOX 78373
Dar Es Salaam, TANZANIA
irb@ihrdc.or.tz
Tel: +255 (0) 22 2774714
Fax: +255(0) 22 2771714

National Institute of Medical Research
P. O. Box 9653
Dar es Salaam
Email; headquarters@nimr.or.tz

27th April 2009

Dr. Abdulnoor Mulokozi
Ifakara Health Institute
P. O. Box 78373
Dar es Salaam

Ref: IHI/IRB/No. A 53

RE: Approval of Protocol Amendment

With reference to your letter dated **6th April 2009**, we here by acknowledge and approve the Protocol entitled; **"Evaluation of the Safety and efficacy of mefloquine as intermittent preventive treatment for malaria in pregnancy"**. The ammendments were approved after the IRB consensus on 14th April 2009.

The main ammendment includes the following:

1. The change in sample size which is 1572 pregnant women per study arm among the 4 sites **(1179 participants/site).** Thus the total sample size to be recruited will be 4716 pregnant women.
2. The measurement of blood pressure at delivery and at un scheduled visits.
3. All women participating in trial 2 will receive CTX prophylaxis as part of the trial intervention
4. The addition of the word "Malaria" in the title of the protocol ***"Evaluation of the Safety and efficacy of mefloquine as intermittent preventive treatment for malaria in pregnancy"***

We advise you to send the same changes to the National Ethical Committee.

Yours' Sincerely

IHI -IRB Chairperson

THE UNITED REPUBLIC OF
TANZANIA

National Institute for Medical Research
P.O. Box 9653
Dar es Salaam
Tel: 255 22 2121400/390
Fax: 255 22 2121380/2121360
E-mail: headquarters@nimr.or.tz
NIMR/HQ/R.8c/Vol. I /78

Ministry of Health and Social Welfare
P.O. Box 9083
Dar es Salaam
Tel: 255 22 2120262-7
Fax: 255 22 2110986

23rd July 2009

Dr Abdunoor Mulokozi
Ifakara Health Institute
P O Box 78373
DAR ES SALAAM

APPROVAL FOR PROTOCOL AMENDMENT

This letter is to confirm that your application for an amendment 01 on the already approved protocol on: Evaluation of the Safety and Efficacy of Mefloquine as intermittent preventive treatment in pregnancy, (Mulokozi A *et al*), Reference NIMR/HQ/R.8a/Vol. IX/804, dated 21st April 2009, has been granted ethics clearance to be conducted in Tanzania.

The Principal Investigator is Dr Abdunoor Mulokozi, IHI, must ensure that the approval is for Amendment 01 Amendment Date is 11th May 2009: To include the changes after investigators meeting in Maputo, Mozambique.

Amendments are:

1. **Evaluation of Mefloquine tolerability**: Women from trial 1 will be randomized to one of the 3 IPTp

   Assumptions for the primary end points: SP vs MQ
   Assumptions for MQ tolerability evaluation: MQ full dose vs MQ split dose

2. **Study procedures:** Agreed only to measure axillary temperature in those women passively reporting symptoms suggestive of malaria, and blood pressure.

3. **Follow up of study children:** Number of scheduled visits of study children should be reduced and a new schedule of follow up to be used.

4. **CTX Prophylaxis:** Clarification is added that all women participating in trial 2 will receive CTX prophylaxis as part of trial intervention.

5. **Other minor changes:** a few minor changes on protocol title, project acronym MiPPAD, Informed Consent Forms according to study design changes (3 arms study). National Policies for Prevention of Mother to Child Transmission of HIV Appendix 8

All changes to the study protocol have been highlighted in bold. The new version of the protocol is dated 27th March 2007. Other conditions remain as per the original approval.

Name: Dr Mwelecele Malecela

Signature

ACTING CHAIRPERSON
MEDICAL RESEARCH
COORDNATING COMMITTEE

Name: Dr Deo Mtasiwa

Signature

CHIEF MEDICAL OFFICER
MINISTRY OF HEALTH
AND SOCIAL WELFARE

RMO
DMO

Corporació Sanitària CLÍNIC

CLÍNIC BARCELONA Hospital Universitari

DICTAMEN DEL COMITÉ ÉTICO DE INVESTIGACIÓN CLÍNICA

Dña. BEGOÑA GÓMEZ PÉREZ, Secretaria del **COMITÉ ÉTICO DE INVESTIGACIÓN CLÍNICA DEL HOSPITAL CLÍNIC I PROVINCIAL DE BARCELONA**

CERTIFICA

Que este Comité ha evaluado la propuesta del promotor, para que se realice:

Amendtment nº 1 dated 27/03/2009

del ensayo clínico:

CÓDIGO: **IP.07.31080.002**
TÍTULO: **"Evaluation of the safety and efficacy of mefloquine as intermittent preventive treatment in pregnacy".**
PROMOTOR: **Fundació Clínic per a la Recerca Biomèdica (FCRB)**

y emite

DICTAMEN FAVORABLE

CIF - G-08431173

Lo que firmo en Barcelona, a 15 de mayo de 2009

Fdo: Dra. Begoña Gómez Pérez

CLÍNIC BARCELONA Hospital Universitari COMITÈ ÈTIC INVESTIGACIÓ CLÍNICA

HOSPITAL CLÍNIC I PROVINCIAL DE BARCELONA
Villarroel, 170 - 08036 Barcelona (España)
Tel. 93 227 54 00 Fax 93 227 54 54
www.hospitalclinic.org

852

CLÍNIC
BARCELONA
Hospital Universitari

DICTAMEN DEL COMITÉ ÉTICO DE INVESTIGACIÓN CLÍNICA

Dña. BEGOÑA GÓMEZ PÉREZ, Secretaria del **COMITÉ ÉTICO DE INVESTIGACIÓN CLÍNICA DEL HOSPITAL CLÍNIC I PROVINCIAL DE BARCELONA**

CERTIFICA

Que este Comité ha evaluado la propuesta del promotor, para que se realice:

Enmienda nº 3 de 15/07/2011 y Protocolo versión 4 de 15/07/2011

del ensayo clínico:

CÓDIGO: **IP.07.31080.002**
TÍTULO: **"Evaluation of the safety and efficacy of mefloquine as intermittent preventive treatment for malaria in pregnacy".**
PROMOTOR: **Fundació Clínic per a la Recerca Biomèdica (FCRB)**

y emite

DICTAMEN FAVORABLE

Y hace constar que:
1º En la reunión celebrada el día 13 de octubre de 2011, acta 18/11 se decidió emitir el informe correspondiente a la enmienda de referencia.
2º En dicha reunión se cumplieron los requisitos establecidos en la legislación vigente – Real Decreto 223/2004 – para que la decisión del citado CEIC sea válida.
3º El CEIC del Hospital Clínic i Provincial, tanto en su composición como en sus PNTs, cumple con las normas de BPC (CPMP/ICH/135/95)
4º La composición actual del CEIC del Hospital Clínic es la siguiente:

Presidente:
Dr. Ramon Gomis de Barbarà (Médico Endocrinólogo, HCB) (Director de Recerca).
Vicepresidente:
Dr. Francisco Javier Carné Cladellas (Médico Farmacólogo Clínico, HCB)
Secretario:
Dra. Begoña Gómez Pérez (Farmacéutica Hospitalaria, HCB)
Vocales:
Prof. Mª Pilar Antón Almerara (Abogada, Observatorio de Bioética y Derecho, UB)
Dra. Marta Aymerich (Médico Hematólogo, HCB)
Sr. Pablo Bassols (Abogado, HCB)
Sra. Montserrat González Creus (Trabajadora Social, Servicio de Atención al Usuario, HCB)
Dra. Montserrat Núñez (Enfermera, HCB)
Dra. Neus Riba García (Farmacóloga Clínica, HCB)
Sr. José Ríos (Estadístico, Farmacología Clínica, USEM, UASP, HCB)
Sr. Octavio Sánchez López (Técnico Auxiliar Clínica, Presidente Asociación Diabéticos de Cataluña)
Dra. José Miguel Sotoca (Farmacéutico Atención Primaria, CAP Les Corts)
Dr. Antoni Trilla García (Médico Epidemiólogo, HCB) (Director UASP)

Que en el caso de que se evalúe algún proyecto del que un miembro sea investigador/colaborador, éste se ausentará de la reunión durante la discusión del proyecto

Lo que firmo en Barcelona, a 14 de octubre de 2011

CLÍNIC BARCELONA Hospital Universitari
COMITÈ ÈTIC INVESTIGACIÓ CLÍNICA

Fdo: Dra. Begoña Gómez Pérez

CIF - G-08431173

CLÍNIC
BARCELONA
Hospital Universitari

DICTAMEN DEL COMITÉ ÉTICO DE INVESTIGACIÓN CLÍNICA

Dña. BEGOÑA GÓMEZ PÉREZ, Secretaria del **COMITÉ ÉTICO DE INVESTIGACIÓN CLÍNICA DEL HOSPITAL CLÍNIC I PROVINCIAL DE BARCELONA**

CERTIFICA

Que este Comité ha evaluado la propuesta del promotor, para que se realice:

Amendment n.2, dated 1st of October 2009, Protocolo versión 3 de 1.10.2009

del ensayo clínico:

CÓDIGO: **IP.07.31080.002**
TÍTULO: **"Evaluation of the safety and efficacy of mefloquine as intermittent preventive treatment for malaria in pregnacy"**
PROMOTOR: **Fundació Clínic per a la Recerca Biomèdica (FCRB)**

y emite

DICTAMEN FAVORABLE

CIF - G-08431173

Lo que firmo en Barcelona, a 17 de diciembre de 2009

CLÍNIC BARCELONA Hospital Universitari COMITÈ ÈTIC INVESTIGACIÓ CLÍNICA

Fdo: Dra. Begoña Gómez Pérez

UNIVERSITE d'ABOMEY - CALAVI
FACULTE DES SCIENCES DE LA SANTE
****
COMITE D'ETHIQUE

01 B.P 188 COTONOU REP. DU BENIN Tél.: (229) 21 30 25 13 Fax : (229) 21304096

Le Président du Comité d'Ethique

N° : 02/10/2009/CE/FSS/UAC

AVIS SUR LE PROTOCOLE « EVALUATION OF SAFETY AND EFFICACY OF MEFLOQUINE AS INTERMITTENT PREVENTIVE TREATMENT IN PREGNANCY / EDCTP-IP.07.31080.002» du 02 mars 2009 dans sa version française

« Evaluation de la tolérance et de l'efficacité de la méfloquine comme traitement préventif intermittent pendant la grossesse. »

Le Comité d'Ethique de la Faculté des Sciences de la Santé réuni en session ordinaire le 29 octobre 2009, a étudié le protocole ci-dessus mentionné.

Les documents suivants ont été examinés et approuvés par le Comité d'Ethique :

- le protocole, **(version française 02 du 27 mars 2009)**
- le formulaire de consentement éclairé, **(version française 02 du 27 mars 2009)**
- le cahier de recueil des données, **(version française 02 du 27 mars 2009)**
- et les annexes 1, 3, 4, 5, 6, 7, 8, 9, 10 et 11.

Le Comité accorde sa clairance éthique à la réalisation de l'étude.

Il convient de prévoir au cours de l'étude et à la charge du promoteur, au moins une visite de supervision sur le terrain de deux membres du Comité.

Cotonou, le 29 Octobre 2009

Le Président

Dr. MEDJI Paul Léon
Professeur d'ORL [illegible]
à la Retraite
C. C. F

Pr MEDJI Ayité P. Léon

Ouma, Peter

From: Kovach, Gloria (CDC/CCID/OD) (CTR)
Sent: Tuesday, March 17, 2009 6:11 PM
To: Erickson, Lynn G. (CDC/CCID/OD) (CTR)
Subject: FW: Protocol #5609: IRB B Restricted Approval (Convened Board - B)

From: Bonds, Constance (CDC/OD/OCSO)
Sent: Tuesday, March 17, 2009 6:43 AM
To: Kovach, Gloria (CDC/CCID/OD) (CTR); Desai, Meghna (CDC/CCID/NCZVED)
Subject: Protocol #5609: IRB B Restricted Approval (Convened Board - B)

DATE: 3/6/2009

FROM: IRB Administrator
Human Research Protection Office
Office of the Chief Science Officer, OD/CDC

[illegible]UBJECT: **IRB Approval of New Protocol #5609**, "An Evaluation of the Safety and Efficacy of Mefloquine as [illegible]termittent Preventive Treatment in Pregnancy" (Convened Board - B)

[illegible]: Meghna Desai [MUD8]
NCZVED/

New protocol #5609 has been approved by CDC IRB "B" for the maximum allowable period of one year and **it will expire on 3/2/2010.**

If other institutions involved in this protocol are being awarded CDC funds through the CDC Procurement and Grants Office (PGO), you are required to send a copy of this IRB approval to the CDC PGO award specialist handling the award. You are also required to verify with the award specialist that the awardee has provided PGO with the required documentation and has approval to begin or continue research involving human subjects as described in this protocol.

As a reminder, the IRB must review and approve all human subjects research protocols at intervals appropriate to the degree of risk, but not less than once per year. There is no grace period beyond one year from the last IRB approval date. It is ultimately your responsibility to submit your research protocol for continuation review and approval by the IRB along with available IRB approvals from all collaborators. Please keep this approval in your protocol file as [illegible]roof of IRB approval and as a reminder of the expiration date. To avoid lapses in approval of your research and the [illegible]ssible suspension of subject enrollment and/or termination of the protocol, please submit your continuation request along with all completed supporting documentation at least six weeks before the protocol's expiration date of [illegible] 2010.

Any problems of a serious nature must be brought to the immediate attention of the IRB, and any proposed changes to the protocol should be submitted as an amendment to the protocol for IRB approval before they are implemented.

If you have any questions, please contact the Human Research Protection Office at (404) 639-4721 or e-mail: huma@cdc.gov.

Constance M. Bonds, BA, MPA
IRB Administrator

cc:
CCID Human Subjects Contact

Katana, Abraham

From: Roberson, Lashonda (CDC/OD/OCSO)
Sent: Tuesday, February 02, 2010 10:23 PM
To: CCID Human Studies Review (CDC)
Subject: FW: CDC IRB Approval of Continuation of Protocol #5609.0, "An Evaluation of the Safety and Efficacy of Mefloquine as Intermittent Preventive Treatment in Pregnancy" (Expedited)

SUBJECT: **CDC IRB Approval of Continuation of Protocol #5609.0**, "An Evaluation of the Safety and Efficacy of Mefloquine as Intermittent Preventive Treatment in Pregnancy" (Expedited)

TO: Meghna Desai, Phd, MPH
CCID/NCZVED/Malaria

CDC's IRB B has reviewed and approved the request to continue protocol #5609.0, " An Evaluation of the Safety and Efficacy of Mefloquine as Intermittent Preventive Treatment in Pregnancy", for the maximum allowable period of one year. **CDC IRB approval will expire on 3/2/2011**. The continuation action was reviewed in accordance with the expedited review process outlined in 45 CFR 46.110(b)(1), categories 2b & 3.

If other institutions involved in this protocol are being awarded CDC funds through the CDC Procurement and Grants Office (PGO), you are required to send a copy of this IRB approval to the CDC PGO award specialist handling the award. You are also required to verify with the award specialist that the awardee has provided PGO with the required documentation and has approval to begin or continue research involving human subjects as described in this protocol.

As a reminder, the IRB must review and approve all human subjects research protocols at intervals appropriate to the degree of risk, but not less than once per year. There is no grace period beyond one year from the last IRB approval date. It is ultimately your responsibility to submit your research protocol for continuation review and approval by the IRB. Please keep this approval in your protocol file as proof of IRB approval and as a reminder of the expiration date. To avoid lapses in approval of your research and the possible suspension of subject enrollment and/or termination of the protocol, please submit your continuation request at least six weeks before the protocol's expiration date of 3/2/2011.

Any problems of a serious nature should be brought to the immediate attention of the IRB, and any proposed changes to the protocol should be submitted as an amendment to the protocol for IRB approval before they are implemented.

If you have any questions, please contact your National Center Human Subjects Contact or the CDC Human Research Protection Office at (404) 639-4721 (or by e-mail at Human Subjects Review - OD on the global CDC global address list or at huma@cdc.gov).

LaShonda Roberson, MPH
LT, USPHS
IRB "B" Administrator
OCSO/OSRS/HRPO
Centers for Disease Control and Prevention
1600 Clifton Rd., NE, Atlanta, GA 30333

cc:

CCID Human Studies Review

Katana, Abraham

From: Desai, Meghna (CDC/CCID/NCZVED) [mud8@cdc.gov]
Sent: Tuesday, February 02, 2010 11:52 PM
To: Ouma, Peter; Katana, Abraham
Subject: FW: 5609: CDC IRB Approval of Continuation of Protocol
Attachments: FW: CDC IRB Approval of Continuation of Protocol #5609.0, "An Evaluation of the Safety and Efficacy of Mefloquine as Intermittent Preventive Treatment in Pregnancy" (Expedited); 1252 v3.0 AMENDMENT.doc; 1254 v1.1 INCIDENT REPORT.doc

FYI

From: Erickson, Lynn G. (CDC/CCID/OD) (CTR)
Sent: Tuesday, February 02, 2010 3:51 PM
To: Desai, Meghna (CDC/CCID/NCZVED)
Cc: CCID Human Studies Review (CDC); NCID DPD Human Subjects (CDC); Fox, LeAnne M. (CDC/CCID/NCZVED); Carr, Wendy (CDC/CCID/OD); Kovach, Gloria (CDC/CCID/OD) (CTR)
Subject: 5609: CDC IRB Approval of Continuation of Protocol

The IRB approval letter for continuation of protocol # 5609 is attached.

<<FW: CDC IRB Approval of Continuation of Protocol #5609.0, "An Evaluation of the Safety and Efficacy of Mefloquine as Intermittent Preventive Treatment in Pregnancy" (Expedited)>>

Please note

This protocol will expire **3/2/2011**.

You may not make any changes to this protocol without obtaining additional IRB approval. The amendment request form has been attached for your convenience.

<<1252 v3.0 AMENDMENT.doc>>

Unanticipated problems involving risks to subjects or others must be **promptly** reported to the IRB. The incident report form has been attached for your convenience.

<<1254 v1.1 INCIDENT REPORT.doc>>

Please contact us if you have any questions.

Lynn Erickson

Human Studies Oversight and Review Team

Strategic Science & Program Unit

Coordinating Center for Infectious Diseases

Centers for Disease Control and Prevention

DEPARTMENT OF HEALTH & HUMAN SERVICES

Public Health Service
Centers for Disease Control
and Prevention (CDC)

Memorandum

Date March 11, 2011

From LaShonda Roberson, MPH
LT, USPHS
IRB-B Administrator, Human Research Protection Office

Subject IRB Approval of Continuation of CDC Protocol #5609, "An Evaluation of the Safety and Efficacy of Mefloquine as Intermittent Preventive Treatment in Pregnancy" (Expedited)

To Meghna Desai, PhD, MPH
CGH/DPDM

CDC's IRB "B" has reviewed and approved your request to continue protocol #5609. The protocol was reviewed in accordance with the expedited review process outlined in 45 CFR 46.110(b) (1), categories 2b and 3.

If other institutions involved in this protocol are being awarded CDC funds through the CDC Procurement and Grants Office (PGO), you are required to send a copy of this IRB approval to the CDC PGO award specialist handling the award. You are also required to verify with the award specialist that the awardee has provided PGO with the required documentation and has approval to begin or continue research involving human subjects as described in this protocol.

As a reminder, the IRB must review and approve all human subjects research protocols at intervals appropriate to the degree of risk, but not less than once per year. There is no grace period beyond one year from the last IRB approval date. It is ultimately your responsibility to submit your research protocol for continuation review and approval by the IRB along with available IRB approvals from all collaborators. Please keep this approval in your protocol file as proof of IRB approval and as a reminder of the expiration date. **To avoid lapses in approval of your research and the possible suspension of subject enrollment and/or termination of the protocol, please submit your continuation request along with all completed supporting documentation at least six weeks before the protocol's expiration date of 03/2/2012.**

Any problems of a serious nature must be brought to the immediate attention of the CDC IRB, and any proposed changes to the protocol should be submitted as an amendment to the protocol for CDC IRB approval before they are implemented.

If you have any questions, please contact your National Center Human Subjects Contact or the CDC Human Research Protection Office (404) 639-4721 or e-mail: huma@cdc.gov.

cc:
CGH Human Subjects

Mal. ✓

-ɔc '-ɔ16

DEPARTMENT OF HEALTH & HUMAN SERVICES

Public Health Service
Centers for Disease Control
and Prevention (CDC)

Memorandum

ate February 19, 2013

rom LaShonda Roberson, MPH
LCDR, USPHS
IRB-B Administrator, Human Research Protection Office

ubject IRB Approval of Continuation of CDC Protocol #5609, "An Evaluation of the Safety and Efficacy of Mefloquine as Intermittent Preventive Treatment in Pregnancy" (Expedited)

o Meghna Desai, PhD, MPH
CGH/DPDM

CDC's IRB "B" has reviewed and approved your request to continue protocol #5609. The protocol was reviewed in accordance with the expedited review process outlined in 45 CFR 46.110(b) (1), categories 2b and 3.

If other institutions involved in this protocol are being awarded CDC funds through the CDC Procurement and Grants Office (PGO), you are required to send a copy of this IRB approval to the CDC PGO award specialist handling the award. You are also required to verify with the award specialist that the awardee has provided PGO with the required documentation and has approval to begin or continue research involving human subjects as described in this protocol.

As a reminder, the IRB must review and approve all human subjects research protocols at intervals appropriate to the degree of risk, but not less than once per year. There is no grace period beyond one year from the last IRB approval date. It is ultimately your responsibility to submit your research protocol for continuation review and approval by the IRB along with available IRB approvals from all collaborators. Please keep this approval in your protocol file as proof of IRB approval and as a reminder of the expiration date. **To avoid lapses in approval of your research and the possible suspension of subject enrollment and/or termination of the protocol, please submit your continuation request along with all completed supporting documentation at least six weeks before the protocol's expiration date of** [redacted]

Any problems of a serious nature must be brought to the immediate attention of the CDC IRB, and any proposed changes to the protocol should be submitted as an amendment to the protocol for CDC IRB approval before they are implemented.

If you have any questions, please contact your National Center Human Subjects Contact or the CDC Human Research Protection Office (404) 639-7570 or e-mail: huma@cdc.gov.

cc:
CGH Human Subjects

Dña. Begoña Gómez Pérez, Secretaria del Comité Ético de Investigación Clínica del Hospital Clínic i Provincial de Barcelona,

CERTIFICA:

Que este Comité, con fecha 09/10/08, ha evaluado la propuesta del promotor para que se realice el ensayo clínico código de protocolo IP.07.31080.002 **titulado "Evaluation of the safety and efficacy of mefloquine as intermittent preventive treatment in pregnacy". Versión 01 de 25/09/08; CI: versión de 25/09/08**, y considera que:

. Se cumplen los requisitos necesarios de idoneidad del protocolo en relación con los objetivos del estudio y están justificados los riesgos y molestias previsibles para el sujeto.

. La capacidad del investigador y los medios disponibles son apropiados para llevar a cabo el estudio.

. Son adecuados tanto el procedimiento para obtener el consentimiento informado como la compensación prevista para los sujetos por daños que pudieran derivarse de su participación en el ensayo.

. El alcance de las compensaciones económicas previstas no interfiere con el respeto a los postulados éticos.

Que este Comité acepta que dicho ensayo clínico sea realizado en el Centro de Investigaçao em Saúde de Manhiça (CISM), Mozambique por la **Dra. Menéndez Santos, Clara** como investigador principal, debiendo ser comunicado a dicho Comité Ético todo cambio en el protocolo o acontecimiento adverso grave.

Lo que firmo en Barcelona, a 17 de octubre de 2008

CLÍNIC
BARCELONA
Hospital Universitari
COMITÈ ÈTIC
INVESTIGACIÓ CLÍNICA

CIF - G-08431173

HOSPITAL CLÍNIC I PROVINCIAL DE BARCELONA
Villarroel, 170 - 08036 Barcelona (España)
Tel. 93 227 54 00 Fax 93 227 54 54
www.hospitalclinic.org

852

KENYA MEDICAL RESEARCH INSTITUTE

KEMRI

KENYA MEDICAL RESEARCH INSTITUTE

P.O. Box 54840 - 00200 NAIROBI, Kenya
Tel: (254) (020) 2722541, 2713349, 0722-205901, 0733-400003; Fax: (254) (020) 2720030
E-mail: director@kemri.org info@kemri.org Website:www.kemri.org

[illegible] INSTITUTE
CENTRE FOR GLOBAL HEALTH RESEARCH
[illegible]
P.O. Box 1578, KISUMU

KEMRI/RES/7/3/1 **November 5, 2009**

TO: **MR. PETER OUMA AND MR. MEGHNA DESAI (PRINCIPAL INVESTIGATORS)**

THROUGH: **DR. J. VULULE, THE DIRECTOR, CGHR, KISUMU**

FORWARDED
9/11/09
DIRECTOR
CENTRE FOR GLOBAL HEALTH RESEARCH

RE: **SSC PROTOCOL No. 1516 (*REQUEST FOR 1ST AMENDMENT*) EVALUATION OF THE SAFETY AND EFFICACY OF MEFLOQUINE AS INTERMITTENT PREVENTIVE TREATMENT IN PREGNANCY (VERSION DATED 20APR2009)**

This is to inform you that during the 171st meeting of KEMRI/National Ethics Review Committee held on Tuesday 13th October 2009, the suggested amendment to the approved study was considered.

The Committee was of the view that the proposed amendment to add two sentences concerning country policies for CTX treatment and measurement of auxiliary temperatures to section 2.5 and the addition of blood pressure measurement in section 2.6 does not alter the risk/benefit status of the study and are granted approval for implementation.

You are required to submit any further amendments to this protocol and other information pertinent to human participation in this study to the SSC and ERC for review prior to initiation.

Yours sincerely,

RCKithinji

R. C. KITHINJI,
FOR: SECRETARY,
KEMRI/NATIONAL ETHICS REVIEW COMMITTEE

KENYA MEDICAL RESEARCH INSTITUTE
CENTRE FOR GLOBAL HEALTH RESEARCH
13 JUL 2009
P.O. Box 1578, KISUMU

KENYA MEDICAL RESEARCH INSTITUTE

P. O . Box 54840 - 00200 NAIROBI, Kenya
Tel: (254) (020) 2722541 , 2713349, 0722-205901, 0733-400003; Fax: (254) (020) 2720030
E-mail: kemri-hq@nairobi.mimcom.net: director @ kemri. org; website: www.kemri.org

KEMRI/RES/7/3/1 **20 MAY 2009**

TO: **MR. PETER OUMA (PRINCIPAL INVESTIGATOR)**

FORWARDED
DIRECTOR
CENTRE FOR GLOBAL HEALTH RESEARCH

THROUGH: **DR. J. VULULE,**
THE DIRECTOR, CGHR,
<u>KISUMU</u>

15/7/09

RE: **SSC PROTOCOL No. 1516 (*REVISED*): EVALUATION OF THE SAFETY AND EFFICACY OF MEFLOQUINE AS INTERMITTENT PREVENTIVE TREATMENT IN PREGNANCY (VERSION DATED 20APR2009)**

Make reference to your letter dated 7 May 2009. We acknowledge receipt of the following documents:

1. The advertising material
2. The Informed Consent Document (ICD) in Dholuo and English
3. The copy of the generic protocol with areas relating to the Kisumu site highlighted

It is now clear how the sample size was arrived at as you have submitted a statement including all the variables and how they fit into the sample size equation. In response to the query about the DSMB it is now apparent that you have no control of who is or is not on the DSMB but that you have suggested to the consortium that a Kenyan be on the DSMB and you are awaiting their response. Kindly, inform the Committee of their response.

The study is hereby granted approval for implementation effective this **20th day of May 2009,** for a period of twelve (12) months.

Please note that authorization to conduct this study will automatically expire on **Tuesday, 19th May 2010.** If you plan to continue with data collection or analysis beyond this date, please submit an application for continuing approval to the ERC Secretariat by **7th April 2010**.

You are required to submit any amendments to this protocol and other information pertinent human participation in this study to the SSC and ERC prior to initiation. You may embark on the study.

Yours sincerely,

RCKithinji

R. C. KITHINJI,
FOR: SECRETARY,
<u>KEMRI/NATIONAL ETHICS REVIEW COMMITTEE</u>

KENYA MEDICAL RESEARCH INSTITUTE

P. O. Box 54840 - 00200 NAIROBI, Kenya
Tel: (254) (020) 2722541 , 2713349, 0722-205901, 0733-400003; Fax: (254) (020) 2720030
E-mail: kemri-hq@nairobi.mimcom.net: director @ kemri. org; website: www.kemri.org

KENYA MEDICAL RESEARCH INSTITUTE
CENTRE FOR GLOBAL HEALTH RESEARCH
13 JUL 2009

KEMRI/RES/7/3/1 **20 MAY 2009**

TO: **MR. PETER OUMA (PRINCIPAL INVESTIGATOR)**

FORWARDED
DIRECTOR
CENTRE FOR GLOBAL HEALTH RESEARCH

THROUGH: **DR. J. VULULE,**
THE DIRECTOR, CGHR,
KISUMU

15/7/09

RE: **SSC PROTOCOL No. 1516 (*REVISED*): EVALUATION OF THE SAFETY AND EFFICACY OF MEFLOQUINE AS INTERMITTENT PREVENTIVE TREATMENT IN PREGNANCY (VERSION DATED 20APR2009)**

Make reference to your letter dated 7 May 2009. We acknowledge receipt of the following documents:

1. The advertising material
2. The Informed Consent Document (ICD) in Dholuo and English
3. The copy of the generic protocol with areas relating to the Kisumu site highlighted

It is now clear how the sample size was arrived at as you have submitted a statement including all the variables and how they fit into the sample size equation. In response to the query about the DSMB it is now apparent that you have no control of who is or is not on the DSMB but that you have suggested to the consortium that a Kenyan be on the DSMB and you are awaiting their response. Kindly, inform the Committee of their response.

The study is hereby granted approval for implementation effective this **20th day of May 2009**, for a period of twelve (12) months.

Please note tha[redacted] this study will automatically expire on Tuesday, 19th [redacted]. If you plan to continue with data collection or analysis beyond this date, please submit an application for continuing approval to the ERC Secretariat by **7th April 2010.**

You are required to submit any amendments to this protocol and other information pertinent human participation in this study to the SSC and ERC prior to initiation. You may embark on the study.

Yours sincerely,

RCKithinji

R. C. KITHINJI,
FOR: SECRETARY,
KEMRI/NATIONAL ETHICS REVIEW COMMITTEE

KENYA MEDICAL RESEARCH INSTITUTE
CENTRE FOR GLOBAL HEALTH RESEARCH
19 MAY 2010

KENYA MEDICAL RESEARCH INSTITUTE

P.O. Box 54840 - 00200 NAIROBI, Kenya
Tel: (254) (020) 2722541, 2713349, 0722-205901, 0733-400003; Fax: (254) (020) 2720030
E-mail: director@kemri.org info@kemri.org Website:www.kemri.org

KEMRI/RES/7/3/1 **May 12, 2010**

TO: **MR. PETER OUMA AND MS. MEGHNA DESAI**
PRINCIPAL INVESTIGATORS

FORWARDED
DIRECTOR
CENTRE FOR GLOBAL HEALTH RESEARCH
20/5/2010

THROUGH: **DR. J. VULULE,**
THE DIRECTOR, CGHR,
KISUMU

RE: **SSC PROTOCOL No. 1516 (*REQUEST FOR ANNUAL RENEWAL)*
EVALUATION OF THE SAFETY AND EFFICACY OF MEFLOQUINE AS INTERMITTENT PREVENTIVE TREATMENT IN PREGNANCY (VERSION DATED 20APR2009)**

Thank you for your Continuing Review Report for the period May 20 2009 to April 22, 2010. This is to inform you that the request for continuation with the above mentioned study was granted expedited review.

The Committee notes that the progress so far includes:
1. The study team was recruited and trained in GCP
2. Procurement of study supplies
3. Establishment of physical infrastructure for the study in collaboration with the Ministry of Health and other KEMRI/CDC research projects
4. The site initiation visit which took place on April 19-22, 2010 by the clinical monitor at the end of which he indicated that the site is ready to commence the study.

The Chair is satisfied that sufficient progress has been made in the review period, and therefore grants the study **provisional approval** to recruit and follow up study participants. This approval is subject to ratification at the 179th meeting of the KEMRI/ERC to be held on June 15, 2010

Please note that **authorization to conduct this study will automatically expire on 11th May 2011.** If you plan to continue with data collection or analysis beyond this date, please submit an application for continuing approval to the ERC Secretariat by **30th March 2011.**

You are required to submit any amendments to this protocol and other information pertinent to human participation in this study to the SSC and ERC for review prior to initiation.

Yours sincerely,

RCKithinji

R. C. KITHINJI,
FOR: SECRETARY,
KEMRI/NATIONAL ETHICS REVIEW COMMITTEE

KENYA MEDICAL RESEARCH INSTITUTE
CENTRE FOR GLOBAL HEALTH RESEARCH

P.O. Box 1578, KISUMU

KENYA MEDICAL RESEARCH INSTITUTE

P.O. Box 54840 - 00200 NAIROBI, Kenya
Tel: (254) (020) 2722541, 2713349, 0722-205901, 0733-400003; Fax: (254) (020) 2720030
E-mail: director@kemri.org info@kemri.org Website:www.kemri.org

KEMRI/RES/7/3/1 **June 24, 2011**

FORWARDED
DIRECTOR
CENTRE FOR GLOBAL HEALTH RESEARCH
22/6/2011

TO: **PETER OKUMA OUMA,**
PRINCIPAL INVESTIGATOR

THRO': **DR. JOHN VULULE,**
THE DIRECTOR, CGHR,
KISUMU

RE: **SSC NO. 1516 (*REQUEST FOR ANNUAL RENEWAL*): EVALUATION OF THE SAFETY AND EFFICACY OF MEFLOQUINE AS INTERMITTENT PREVENTIVE TREATMENT OF MALARIA IN PREGNANCY.**

This is to inform that during the 190th meeting of the KEMRI/ERC meeting held on the 14th of June 2011, the Committee **conducted the annual review and approved** the above referenced application for another year.

We acknowledge receipt of the following documents:

1. Study status report dated 6 April 2011-06-23
2. MiPPAD protocol version 3 dated October 1, 2009
3. Site specific document version 5 dated January 7, 2010
4. Informed Consent Document (ICD) English, Kiswahili and Dholuo

The Committee notes that 236 women have been enrolled in the main study and provided with appropriate study intervention and clinical follow up, a further 51 have been enrolled for the ancillary study (amendment 4) and overall 196 blood spots samples collected for analysis. In view of the current enrolment rate of 2 women per month it is anticipated that enrolment will be complete by September 2011. Future plans are therefore to follow up study subjects.

This approval is valid from today **June 14, 2011** through to **June 14, 2012**. Please note that authorization to conduct this study will automatically expire on **June 14, 2012.**

If you plan to continue with data collection or analysis beyond this date please submit an application for continuing approval to the ERC secretariat by **April 14, 2012.**

You are required to submit any amendments to this protocol and other information pertinent to human participation in this study to the SSC and ERC for review prior to initiation.

Yours sincerely,

ROKithinji

Caroline Kithinji,
FOR: Secretary
KEMRI/ETHICS REVEIW COMMITTEE

KENYA MEDICAL RESEARCH INSTITUTE

P.O. Box 54840-00200, NAIROBI, Kenya
Tel (254) (020) 2722541, 2713349, 0722-205901, 0733-400003; Fax: (254) (020) 2720030
E-mail: director@kemri.org info@kemri.org Website:www.kemri.org

KEMRI/RES/7/3/1 **July 11, 2012**

TO: **DR. MEGHNA DESAI (PRINCIPAL INVESTIGATOR)**

THROUGH: **DR. JOHN VULULE,**
THE DIRECTOR, CGHR,
KISUMU

Dear Madam,

RE: SSC PROTOCOL No. 1516 (*RATIFICATION OF CONTINUATION APPROVAL*): EVALUATION OF THE SAFETY AND EFFICACY OF MEFLOQUINE AS INTERMITTENT PREVENTIVE TREATMENT OF MALARIA IN PREGNANCY

This is to inform you that at the 204th meeting of the KEMRI Ethics Review Committee held on 10th July 2012, the Committee ratified the continuation approval granted by the ERC Chair on **July 4, 2012**.

Please note that authorization to conduct this study will automatically expire on **July 3, 2013**. If you plan to continue with data collection or analysis beyond this date, please submit an application for continuing approval to the ERC Secretariat by **May 22, 2013**.

You are required to submit any proposed changes to this study to the SSC and ERC for review and the changes should not be initiated until written approval from the ERC is received. Please note that any unanticipated problems resulting from the conduct of this study should be brought to the attention of the ERC and you should advise the ERC when the study is completed or discontinued.

You may continue with the study.

DR. CHRISTINE WASUNNA,
ACTING SECRETARY,
KEMRI ETHICS REVIEW COMMITTEE

KENYA MEDICAL RESEARCH INSTITUTE
CENTRE FOR GLOBAL HEALTH RESEARCH
30 OCT 2013
P.O. Box 1578, KISUMU

KENYA MEDICAL RESEARCH INSTITUTE

P.O. Box 54840-00200, NAIROBI, Kenya
Tel (254) (020) 2722541, 2713349, 0722-205901, 0733-400003; Fax: (254) (020) 2720030
E-mail: director@kemri.org info@kemri.org Website:www.kemri.org

KEMRI/RES/7/3/1 **October 22, 2013**

TO: **DR. MEGHNA DESAI**
PRINCIPAL INVESTIGATOR

THRO': **DR. STEPHEN MUNGA**
ACTING DIRECTOR, CGHR,
KISUMU

FORWARDED
DIRECTOR
CENTRE FOR GLOBAL HEALTH RESEARCH

30/10/2013

Dear Madam,

RE: **SSC NO. 1516 (*REQUEST FOR ANNUAL RENEWAL*): EVALUATION OF THE SAFETY AND EFFICACY OF MEFLOQUINE AS INTERMITTENT PREVENTIVE TREATMENT OF MALARIA IN PREGNANCY**

Thank you for the continuing review report for the period **4th July 2012** to **September 2013.**

This is to inform you that during the 220th meeting of the KEMRI/ERC held on **22nd October 2013**, the Committee **conducted the annual review and approved** the above referenced application for another year.

This approval is valid from today 22nd October 2013 through to 21st October 2014. Please note that authorization to conduct this study will automatically expire on **21st October 2014**. If you plan to continue with data collection or analysis beyond this date please submit an application for continuing approval to the **ERC** secretariat by **9th September 2014.**

You are required to submit any amendments to this protocol and other information pertinent to human participation in this study to the SSC and ERC for review prior to initiation.

Yours faithfully,

EAB

DR. ELIZABETH BUKUSI,
ACTING SECRETARY,
KEMRI/ETHICS REVIEW COMMITTEE

KENYA MEDICAL RESEARCH INSTITUTE
CENTRE FOR GLOBAL HEALTH RESEARCH
24 MAR 2009
P.O. Box 1578, KISUMU

KENYA MEDICAL RESEARCH INSTITUTE

P.O. Box 54840 - 00200 NAIROBI, Kenya
Tel: (254) (020) 2722541, 2713349, 0722-205901, 0733-400003; Fax: (254) (020) 2720030
E-mail: kemri-hq@nairobi.mimcom.net: director@kemri.org; Website: www.kemri.org

ESACIPAC/SSC/4334

20th March, 2009

FORWARDED
DIRECTOR
CENTRE FOR GLOBAL HEALTH RESEARCH
[illegible] 3/09

Peter Ouma

Thro'

Director, CGHR
P.O. Box 1578
KISUMU

REF: SSC No.1516 (Revised) – Evaluation of the safety and efficacy of mefloquine as intermittent preventive treatment in pregnancy

I am pleased to inform you that the above mentioned proposal, in which you are the PI, was discussed by the KEMRI Scientific Steering Committee (SSC), during its **154th meeting** held on **3rd March 2009** and has since been approved for implementation by the SSC.

The SSC however, advises that work on this project can only start when ERC approval is received.

[signature]

C. Mwandawiro, PhD
SECRETARY, SSC

KENYA MEDICAL RESEARCH INSTITUTE

P.O. Box 54840 - 00200 NAIROBI, Kenya
Tel: (254) (020) 2722541, 2713349, 0722-205901, 0733-400003; Fax: (254) (020) 2720030
E-mail: director@kemri.org info@kemri.org Website:www.kemri.org

KEMRI/RES/7/3/1 **July 8, 2010**

TO: **MR. PETER OUMA AND MR. MEGHNA DESAI**
PRINCIPAL INVESTIGATORS

THROUGH: **DR. J. VULULE,**
THE DIRECTOR, CGHR,
KISUMU

RE: **SSC PROTOCOL No. 1516 (*REQUEST FOR 2ND AMENDMENT)***
EVALUATION OF THE SAFETY AND EFFICACY OF MEFLOQUINE AS INTERMITTENT PREVENTIVE TREATMENT IN PREGNANCY (VERSION DATED 20APR2009)

This is to inform you that your request for an amendment to the approved study was granted expedited review.

The Committee was of the view that the proposed amendments do not alter the risk/benefit status of the study and are granted approval for implementation.

You are required to submit any further amendments to this protocol and other information pertinent to human participation in this study to the SSC and ERC for review prior to initiation.

Yours sincerely,

RCKithinji

R. C. KITHINJI,
FOR: SECRETARY,
KEMRI/NATIONAL ETHICS REVIEW COMMITTEE

KENYA MEDICAL RESEARCH INSTITUTE
CENTRE FOR GLOBAL HEALTH RESEARCH
30 NOV 2011
P.O. Box 1578, KISUMU

KENYA MEDICAL RESEARCH INSTITUTE

P.O. Box 54840-00200, NAIROBI, Kenya
Tel (254) (020) 2722541, 2713349, 0722-205901, 0733-400003; Fax: (254) (020) 2720030
E-mail: director@kemri.org info@kemri.org Website:www.kemri.org

KEMRI/RES/7/3/1 **NOVEMBER 22, 2011**

TO: **MR. PETER OUMA AND ~~MR. MEGHNA DESAI~~**
PRINCIPAL INVESTIGATORS

FORWARDED
DIRECTOR
CENTRE FOR GLOBAL HEALTH RESEARCH

THROUGH: **DR. J. VULULE,**
THE DIRECTOR, CGHR,
KISUMU

RE: **SSC PROTOCOL No. 1516 (*REQUEST FOR 6TH AMENDMENT)* EVALUATION OF THE SAFETY AND EFFICACY OF MEFLOQUINE AS INTERMITTENT PREVENTIVE TREATMENT IN PREGNANCY (*VERSION DATED 20APR2009*)**

This is to inform you that during the 195th meeting of the KEMRI/ERC meeting held on November 22, 2011, the requested amendment for the above referenced study was reviewed.

The Committee noted the following amendments:

a. Study protocol EDCTP- IP.07.31080.002 (version 04, 15 July 2011)
b. Amendment 03 (15 July 2011)
c. Site-specific addendum (version 07, 22 July 2011)
d. Informed consent-English (version 02, 22 July 2011)
e. Informed consent-Swahili (version 02, 22 July 2011)
f. Informed consent-Dholuo (version 02, 22 July 2011)

The following are the proposed changes:

1. In reference to the Sponsor's (main) protocol A (protocol version 04 and amendment 03), the following revisions are proposed:
   a. To include information currently available in the study standard operating procedures that is considered important to be included in the protocol.
   b. Incorporate the corrected lists of trials endpoints in the study protocol to be consistent with study analysis plans

b. Incorporate the corrected lists of trials endpoints in the study protocol to be consistent with study analysis plans

c. Update other changes such as revised list of investigators, addition of the study flow as an annex.

2. To increase the sample size for the KEMRI/CDC site from 360 HIV-infected pregnant women to 465 due to slow recruitment at the Tanzanian site. This adjustment would ensure that a total of 1070 HIV-infected women are enrolled as initially planned so that the study is sufficiently powered.

3. The provision to ship placental tissue blocks and blood smears for quality control (QC) in Barcelona (Spain). The standard operating procedure for the study stipulates that all participating sites in this multi-centre trial should ship samples for QC to one central laboratory. The amendment for this arrangement (section 2.12 on page 12 of 14 of the site specific document) has previously been approved by the ERC but the informed consent document now amended to accommodate this change.

The suggested amendment is justified however kindly specify in the consent documents where exactly in Barcelona, Spain the samples will be sent for the purposes of QC. Is it to Barcelona Centre for International Health Research (CRESIB)?

Yours sincerely,

RCKithinji

Caroline Kithinji
FOR: SECRETARY,
KEMRI/ETHICS REVIEW COMMITTEE

REPÚBLICA DE MOÇAMBIQUE

MINISTÉRIO DA SAÚDE

COMITÉ NACIONAL DE BIOÉTICA PARA A SAÚDE
IRB00002657

Exmo Senhor
Dr. Eusébio Macete
CISM

Ref: 282/CNBS/11 **Data 29 de Setembro de 2011**

Assunto: *aprovação da emenda 3 do protocolo "Avaliação da segurança e eficácia da mefloquina como tratamento preventivo da malária durante a gravidez (MIPPAD)."*

O Comité Nacional de Bioética para a Saúde (CNBS) analisou o pedido de aprovação da emenda 3 de 8 de Agosto de 2011 do protocolo intitulado: ***"Avaliação da segurança e eficácia da mefloquina como tratamento preventivo da malária durante a gravidez (MIPPAD)."*** **Sobre o mesmo chegou a seguinte conclusão:**

O CNBS não vê nenhum inconveniente de ordem ética que impeça a introdução da emenda pelo que, dá a sua devida autorização.

Contudo, recomenda que os investigadores que o mantenham informado do decurso do estudo.

Faz notar que a aprovação ética não substitui a autorização administrativa.

Sem mais de momento, cordiais saudações.

O Vice-Presidente

Dr. João Schwalbach

REPÚBLICA DE MOÇAMBIQUE MINISTÉRIO DA SAÚDE COMITÉ NACIONAL DE BIOÉTICA PARA A SAÚDE

ENDEREÇO:
MINISTÉRIO DA SAÚDE
C. POSTAL 264
Av. Eduardo Mondlane/Salvador Allende
MAPUTO – MOÇAMBIQUE

Telefones: 430814/427131(4)
Telex: 6-239 MISAU MO
FAX: 258 (1) 426547
258 (1) 33320

REPÚBLICA DE MOÇAMBIQUE

MINISTÉRIO DA SAÚDE

COMITÉ NACIONAL DE BIOÉTICA PARA A SAÚDE
IRB00002657

Exmo Senhor
Dr. Eusébio Macete
CISM

Ref: 282/CNBS/11 **Data 29 de Setembro de 2011**

Assunto: *aprovação da emenda 3 do protocolo "Avaliação da segurança e eficácia da mefloquina como tratamento preventivo da malária durante a gravidez (MIPPAD)."*

O Comité Nacional de Bioética para a Saúde (CNBS) analisou o pedido de aprovação da emenda 3 de 8 de Agosto de 2011 do protocolo intitulado: ***"Avaliação da segurança e eficácia da mefloquina como tratamento preventivo da malária durante a gravidez (MIPPAD)."*** **Sobre o mesmo chegou a seguinte conclusão:**

O CNBS não vê nenhum inconveniente de ordem ética que impeça a introdução da emenda pelo que, dá a sua devida autorização.

Contudo, recomenda que os investigadores que o mantenham informado do decurso do estudo.

Faz notar que a aprovação ética não substitui a autorização administrativa.

Sem mais de momento, cordiais saudações.

O Vice-Presidente

Dr. João Schwalbach

ENDEREÇO:
MINISTÉRIO DA SAÚDE
C. POSTAL 264
Av. Eduardo Mondlane/Salvador Allende
MAPUTO – MOÇAMBIQUE

Telefones: 430814/427131(4)
Telex: 6-239 MISAU MO
FAX: 258 (1) 426547
258 (1) 33320

REPÚBLICA DE MOÇAMBIQUE

MINISTÉRIO DA SAÚDE

COMITÉ NACIONAL DE BIOÉTICA PARA A SAÚDE

Exma Senhora
Drª Clara Menéndez, MD,PhD

Ref.147 /CNBS Data 8 de Maio de 2008

Assunto: Parecer sobre o estudo "Avaliação da segurança e eficácia da mefloquina como tratamento preventivo da malária durante a gravidez".

Reunido no dia 07 de Maio de 2009 o Comité Nacional de Bioética para a Saúde (CNBS) analisou as modificações efectuadas ao protocolo intitulado: "**Avaliação da segurança e eficácia da mefloquina como tratamento preventivo da malária durante a gravidez**", sobre o mesmo o CNBS chegou a seguinte conclusão:

O CNBS não vê nenhum inconveniente de ordem ética que impeça a realização do estudo pelo que, dá a sua devida aprovação.

Contudo, recomenda aos investigadores que o mantenham informado do decurso do estudo.

Faz notar que a aprovação ética não substitui a autorização administrativa.

Sem mais de momento as nossas cordiais saudações.

O Presidente

Dr. João Manuel de Carvalho Fumane

REPÚBLICA DE MOÇAMBIQUE MINISTÉRIO DA SAÚDE COMITÉ NACIONAL DE BIOÉTICA PARA A SAÚDE

ENDEREÇO:
MINISTÉRIO DA SAÚDE
C. POSTAL 264
Av. Eduardo Mondlane/Salvador Allende
MAPUTO – MOÇAMBIQUE

Telefones: 430814/427131(4)
Telex: 6-239 MISAU MO
FAX: 258 (1) 426547
258 (1) 33320

REPÚBLICA DE MOÇAMBIQUE

MINISTÉRIO DA SAÚDE

Gabinete do Ministro

Exmo Senhor
Director do CIS Manhiça
Dr. Eusébio Macete

Nota n.º 2008 / 004/GMS/09 Maputo, 13 de Julho de 2009

Assunto: Solicitação de autorização para início de estudo.

Incumbe-me Senhor Ministro da Saúde, Prof. Dr. Paulo Ivo Garrido, de acusar a recepção da Nota n.º. 1999/03, de 07.07.2009, na qual solicitam a autorização para início de estudo com o título: " **Avaliação da segurança e eficácia da Mefloquina (MQ) como tratamento intermitente preventivo de malária durante a gravidez**", e, tenho a informar o despacho recaído cujo o teor é o seguinte:

"Autorizo."
Assinado: Prof. Dr. Paulo Ivo Garrido
(10/07/2009)

Cumprimentos.

O Chefe do Gabinete

Tiágo Macuácua

Av. Eduardo Mondlane / Salvador Allende
Caixa Postal Nº 264
E-mail: gbminsau@tropical.co.mz
Maputo – República de Moçambique

Telefs.: 21 31 44 88 - 21 30 66 21
21 42 26 82
Telefax: 258 (21) 42 71 33
Telex: 6-239 MISSAU MO

REPÚBLICA DE MOÇAMBIQUE

MINISTÉRIO DA SAÚDE

COMITÉ NACIONAL DE BIOÉTICA PARA A SAÚDE

Exma Senhora
Dr.ª Clara Menéndez, MD, PhD

Ref.203/CNBS

Data 23 de Junho de 2009

Assunto: Parecer sobre o estudo "Avaliação da segurança e eficácia da mefloquina como tratamento preventivo da malária durante a gravidez."

O Comité Nacional de Bioética para a Saúde (CNBS) analisou a emenda 1 ao protocolo intitulado: **"Avaliação da segurança e eficácia da mefloquina como tratamento preventivo da malária durante a gravidez."** sobre o mesmo chegou a seguinte conclusão:

O CNBS não vê nenhum inconveniente de ordem ética que impeça a realização do estudo pelo que, dá a sua devida aprovação.

Contudo, recomenda aos investigadores que o mantenham informado do decurso do estudo.

Faz notar que a aprovação ética não substitui a autorização administrativa.

Sem mais de momento as nossas cordiais saudações.

O Presidente

Dr. João Manuel de Carvalho Fumane

REPÚBLICA DE MOÇAMBIQUE MINISTÉRIO DA SAÚDE COMITÉ NACIONAL DE BIOÉTICA PARA A SAÚDE

ENDEREÇO:
MINISTÉRIO DA SAÚDE
C. POSTAL 264
Av. Eduardo Mondlane/Salvador Allende
MAPUTO – MOÇAMBIQUE

Telefones: 430814/427131(4)
Telex: 6-239 MISAU MO
FAX: 258 (1) 426547
258 (1) 33320

REPÚBLICA DE MOÇAMBIQUE

MINISTÉRIO DA SAÚDE

COMITÉ NACIONAL DE BIOÉTICA PARA A SAÚDE

IRB 00002657

Exma Senhora
Dr.ª Clara Menéndez, MD,PhD
CISM

Ref.12/CNBS

Data 28 de Janeiro de 2010

Assunto: Parecer sobre *"Avaliação da segurança e eficácia da mefloquina como tratamento preventivo da malária durante a gravidez."*

O Comité Nacional de Bioética para a Saúde (CNBS) analisou o pedido de aprovação da emenda 2 de 1 de Outubro de 2009 do protocolo intitulado: ***"Avaliação da segurança e eficácia da mefloquina como tratamento preventivo da malária durante a gravidez."***, sobre o mesmo o CNBS chegou a seguinte conclusão:

Não havendo nenhum inconveniente de ordem ética que impeça a introdução da emenda do estudo, o CNBS dá a sua devida aprovação.

Recomenda aos investigadores que o mantenham informado do decurso do estudo.

E faz notar que a aprovação ética não substitui a autorização administrativa.

Sem mais de momento as nossas cordiais saudações.

O Presidente

Dr. João Man[illegible] Carvalho Fumane

ENDEREÇO:
MINISTÉRIO DA SAÚDE
C. POSTAL 264
Av. Eduardo Mondlane/Salvador Allende
MAPUTO – MOÇAMBIQUE

Telefones: 430814/427131(4)
Telex: 6-239 MISAU MO
FAX: 258 (1) 426547
258 (1) 33320

IFAKARA HEALTH INSTITUTE
research | training | services

INSTITUTIONAL REVIEW BOARD
P O BOX 78373 DAR ES SALAAM, TANZANIA
Tel +255 (0) 22 2774714, Fax: + 255 (0) 22 2771714 Email: irb@ihi.or.tz

National Institute for Medical Research
P O Box 9653
Dar Es Salaam
Email: headquarters@nimr.or.tz

12th August, 2011

Abdunoor Mulokozi
Ifakara Health Institute
P O Box 78373
Dar Es Salaam

IHI/IRB/AMM 15 – 2011

AMMENDMENT APPROVAL

On 11th August 2011, the Ifakara Health Institute Review Board (IHI IRB) approved Ammendment 3 of a project titled: *"Evaluation of the safety and efficacy of Mefloquine as Intermittent Preventive Treatment for Malaria in Pregnancy"* submitted by the Principal Investigator Abdunoor Mulokozi.

Ammendment 3 changes included in submitted protocol dated 1st August 2011 include:

- Dr Mwaka Athman Kakolwa has been added to Tanzania site
- Tanzania site moved from Ifakara to Dodoma
- Changes on statements regarding study implementation procedures (pg 14, pg 23, pg 15, pg 16, pg 13, pg 22)
- List of trial end points in the study protocol (pg 13 and pg 22) have been corrected to be consistent with study analysis plans
- Changes to time points of blood samples collection onto filter papers (for PCR analysis on pg 19 and pg 29)
- The trial flow chart has been added as on appendix 12 (pg 82 and pg 83)

The IRB reserves the right to undertake field inspections to check on the protocol compliance

IRgalli 30th Nov 2011

Secretary
BEVERLY MSAMBICHAKA

INSTITUTIONAL REVIEW BOARD
P O BOX 78373 DAR ES SALAAM, TANZANIA
Tel +255 (0) 22 2774714, Fax: + 255 (0) 22 2771714 Email: irb@ihi.or.tz

National Institute for Medical Research
P O Box 9653
Dar Es Salaam
Email; headquarters@nimr.or.tz

28th Jan 2010

Abdulnoor Mulokozi
Ifakara Health Institute
P O Box 78373
Dar Es Salaam

Ref: IHI/IRB/AMM/02 – 2010

AMMENDMENT 2 APPROVAL

On 28th January 2010, the Ifakara Health Institute Review Board (IHI IRB) reviewed an amendment to a study titled: "**Evaluation of the Safety and Efficacy of Mefloquine as Intermittent Preventive Treatment for Malaria in Pregnancy",** submitted by the Principal Investigator Abdulnoor Mulokozi . The study with previous approval number IHI/IRB/No 53 dated 10th December 2009.

The following changes have been approved following IRB Consensus:

1. Rephrasing of study hypothesis 4: … addition of MQ-IPTp to cotrimoxazole prophylaxis is ***more*** efficacious ***than*** cotrimoxazole alone…
2. Rearranging study second secondary objective, now listed as a primary objective
3. Design of Trial 2 to include the term "Superiority Clinical Trial"
4. The number of stillbirths has been added as a secondary endpoint of trial 1 and trial 2.
5. The wording of the inclusion procedures of the study participants has been changed: ….will be ***included into the study and will be asked to sign the informed consent*** if ……
6. Following DSMB suggestion, all study participants will be observed for 60 minutes be IPTp administration
7. Following DSMB suggestion, study investigators should notify all SAEs (including deaths) within 48hrs)
8. In section 5.4 page 32 of study protocol, the word "excluded" has been changed to "withdrawn"
9. In section 9.4 of study protocol it has been added that MQ efficacy to prevent LBW is deemed to be equal in the 2MQ groups(full dose and split dose)
10. Follow up of study participants has been extended until one month after delivery
11. ICF (Appendix 2) of study participants from trial 1 has been amended, Fansidar has been replaced with SP.
12. Names of study investigators has in page 3 have been updated as of 1st October 2009

The Principal Investigator of the study must ensure:

1. PI should submit a six month progress report and the final report at the end of the project
2. Any amendment, which will be done after the approval of the protocol, must be communicated as soon as possible to the IRB for another approval
3. All research must stop after the project expiration date, unless there is prior information and justification to the IRB.
4. There should be plans to give feedback to the community on the findings
5. Any publication needs to pass through the IRB

The IRB reserves the right to undertake field inspections to check on the protocol compliance

Chairperson
JOYCE K. IKINGURA

Administrator
BEVERLY L. MSAMBICHAKA

IFAKARA HEALTH INSTITUTE
research | training | services

INSTITUTIONAL REVIEW BOARD

P.O.BOX 78373
Dar Es Salaam, TANZANIA
irb@ihrdc.or.tz
Tel: +255 (0) 22 2774714
Fax: +255(0) 22 2771714

National Institute of Medical Research
P. O. Box 9653
Dar es Salaam
Email; headquarters@nimr.or.tz

10th December 2008

Dr. Abdunoor Mulokozi
Ifakara Health Institute (IHI)
P. O. Box 78373
Dar es Salaam

IHRDC/IRB/No. A 53

INSTITUTIONAL CLEARANCE CERTIFICATE FOR CONDUCTING HEALTH RESEARCH

On 29th November 2008, the Ifakara Health Institute Institutional Review Board (IHI IRB) reviewed the study entitled: **"Evaluation of the Safety and Efficacy of Mefloquine as intermittent preventive treatment in Pregnancy".** Submitted by the Principle Investigator Dr. Abdunoor Mulokozi

The following documents were reviewed:

1. Protocol -
2. Informed Consent Forms
3. Questionnaires
4. CVs

The study has been approved for implementation after IRB consensus.

This certificate thus indicates that; the above-mentioned study has been granted an Institutional ethics clearance to conduct the above named study in Ifakara, Kilombero District.

The Principal Investigator of the study must ensure that, the following conditions are fulfilled during or after the implementation of the study:

1. PI should submit a six month progress report and the final report at the end of the project
2. **Any amendment**, which will be done after the approval of the protocol, must be communicated as soon as possible to the IRB for another approval.
3. All research must stop after the project expiration date, unless there is prior information and justification to the IRB.
4. There should be plans to give feedback to the community on the findings
5. Any publication needs to pass through the IRB.

The IRB reserves the right to undertake field Inspections to check on the protocol compliance

Joyce K. Ikingura

Signature
Chairperson

Sally Ntenga

Signature
Administrator
